# Current evidence on the core components of the geriatric rehabilitation process: a systematic review

**DOI:** 10.1007/s40520-026-03450-z

**Published:** 2026-07-15

**Authors:** Martin Skoumal, Martina Honegger, Stefan Grund, Petra Benzinger, Stefan Bachmann, Thomas Münzer, Stefan Goetz, Thomas E. Dorner, Christoph Pertinatsch, Britta Neubacher, Christof Kadane, Sonja Lindner-Rabl, Regina Roller-Wirnsberger

**Affiliations:** 1 Department of Internal Medicine, Medical University of Graz, Research Unit Services for Old Age and Life Long Health, Graz, Austria; 2 Department for Scientific Research in Rehabilitation, Pension Insurance Austria, Vienna, Austria; 3 Geriatric Center, Medical Faculty Heidelberg, Heidelberg University, Heidelberg, Germany; 4 Klinikgruppe Valens, Valens, Switzerland; 5 Department of Geriatrics, Inselspital, Universitätsspital Bern, Bern, Switzerland; 6 Geriatrische Klinik, Sankt Gallen, Switzerland; 7 Spital Thurgau, Thurmed AG, Frauenfeld, Switzerland; 8 Academy of Ageing Research, Haus Der Barmherzigkeit, Vienna, Austria; 9 Centre for Public Health, Department of Social and Preventive Medicine, Medical University Vienna, Vienna, Austria; 10 Swiss Society of Physical Medicine and Rehabilitation, Steinhausen, Switzerland; 11 Faculty of Medical Sciences, Private University in the Principality of Liechtenstein (UFL), Triesen, Principality of Liechtenstein

**Keywords:** Geriatric rehabilitation care process, Older person, participation, Comprehensive Geriatric Assessment

## Abstract

**Background:**

Geriatric rehabilitation (GR) is a key component of integrated care for older persons with multimorbidity, yet its fundamental care process and core components that enable effective GR remain poorly defined and highly variable.

**Methods:**

We conducted a systematic review (Registration ID: PROSPERO 2025 CRD42025642761) to identify and synthesise evidence on core components of the GR care process. Medline, CINAHL, Cochrane and Embase were searched to 31 January 2025. Randomised controlled trials (RCTs) and controlled prospective cohort studies involving multimorbid geriatric patients undergoing multidisciplinary GR programmes were included. Study quality was assessed using the JBI critical appraisal tool.

**Results:**

Thirty-six studies (31 RCTs and 5 controlled prospective cohort studies), comprising 10,647 patients, met the inclusion criteria. Studies represented a broad range of GR service types. Substantial heterogeneity was observed in reported care processes, including terminology, team composition, workflow, contextual factors, and assessment methods, with over 90 different tools used. Comprehensive geriatric assessment (CGA) was rarely reported, patients with moderate to severe dementia were frequently excluded, and no study described the entire GR care process in a holistic manner.

**Discussion:**

Current evidence shows substantial heterogeneity and fragmentation in the description and implementation of the GR care process, with variable depth of implementation, team structures and assessments. The lack of reproducible process structures and clearly defined core components limits comparability, implementation, and evaluation.

**Conclusion:**

Future research should adopt study designs spanning the full GR pathway across settings, incorporate standardised process indicators, a toolbox of CGA and goal-setting instruments and evaluate outcomes aligned with the biopsychosocial principles of the ICF.

**Supplementary Information:**

The online version contains supplementary material available at https://doi.org/10.1007/s40520-026-03450-z.

## Introduction

Rehabilitation is recognised as an essential health service within the World Health Organization (WHO) Rehabilitation initiative, which calls for improved governance, workforce capacity and service delivery across the care continuum [1, 2]. For ageing societies, these priorities are especially pressing, underscoring the importance of geriatric rehabilitation (GR) as a key component of integrated care for older adults with multiple health conditions and geriatric syndromes [3–5]. GR is defined as a multidimensional approach of diagnostic and therapeutic interventions, with the primary goal to improve patients’ functional abilities and participation in daily life [6, 7]. Gordon et al. have recently argued that establishing a right to GR is critical to ensuring equitable and timely care for older adults with frailty, multimorbidity or post-acute functional decline, and that achieving this requires embedding robust system-level quality standards, interdisciplinary collaboration and sustained policy engagement [8]. Earlier systematic reviews conducted in 2010 by Bachmann et al. and in 2024 by Wong et al. show that GR can improve outcomes related to functioning, admission to nursing homes, and mortality [9, 10]. A recent systematic review confirms the predictive role of overall intrinsic capacity showing that declines in locomotion are a key driver of functional impairment [11]. Existing evidence shows that GR is multiprofessional and team-based, ideally guided by the biopsychosocial model as proposed in the International Classification of Functioning, Disability, and Health (ICF) [12]. Yet, its organisation and availability remain highly variable across health systems and settings [13]. Prior reviews highlighted the following key challenges: triage decisions for GR rely on heterogeneous clinical and psychosocial factors, with no consensus on a core assessment set [14], outpatient and home-based GR programmes demonstrate comparable outcomes, but differ substantially in structure, timing, coordination and interdisciplinary involvement [15]. Community-based complex interventions show benefits for independent living and mortality but vary widely in composition and implementation [16]. Moreover, implementation studies underscore that many complex interventions achieve only partial uptake due to inconsistent processes, unclear team roles and lack of standardisation [17].

These findings indicate that while GR can improve functional outcomes, support independent living and reduce mortality, the evidence on core components of the GR care process is poorly described and inconsistently implemented. This limits comparability across care models and studies and leaves health systems without a coherent framework to organise and/or evaluate GR services. To address this critical gap, we conducted a systematic review to identify and synthesise existing evidence on the core components of a person-centred GR process.

## Methods

### Protocol and registration

The methodology for this systematic review followed the Preferred Reporting Items for Systematic Reviews and Meta-Analyses (PRISMA) 2020 guidelines [18]. A review protocol specifying the objectives, eligibility criteria, search strategy, screening approach and analysis plan was developed a priori and registered in PROSPERO (Registration ID: (PROSPERO 2025 CRD42025642761) and is available from URL: https://www.crd.york.ac.uk/prospero/display_record.php?ID=CRD42025642761.

### Inclusion criteria

Studies were eligible if they met the following criteria, according to the PICOS scheme [19]: Population: Adults aged ≥ 65 years or mean age 75 years with multimorbidity and/or frailty enrolled in GR programmes according to the definition of the 2020 European consensus on core principles and future priorities for GR consensus statement [6]. Intervention: Multiprofessional, coordinated GR programmes in various settings (e.g. geriatric inpatient, outpatient (ambulatory, day rehabilitation) home-based, mobile GR, geriatric telerehabilitation). Comparison: Usual care (depending on study design). Outcomes: Empirical descriptions of GR patient-centred core components of care processes, and workflow organisation as well as process-related quality elements within GR to maximise patients’ independence in activities of daily living (ADL), quality of life (QoL) and self-care capability. Primary outcomes comprised reporting of process-related core components, structures and organisational features of GR such as eligibility criteria for admission, recommended measurements/assessments (e.g. comprehensive geriatric assessment (CGA)), patient-centred goal-setting, multiprofessional team structure, treatment process including personalised therapy planning, discharge and care transition management.

Secondary outcomes included facilitators and barriers, specific GR process and setting requirements (e.g. length of stay), education and resources in GR as well as mortality.

Study design: We included randomised controlled trials (RCTs) and controlled prospective cohort studies because the review aimed to identify care-process components supported by comparative outcome evidence, rather than to catalogue all described rehabilitation processes.

### Exclusion criteria

Studies that focused solely on effectiveness without reporting process components, single-discipline interventions, and interventions that were not part of a rehabilitation programme, populations younger than 65 years, and non-empirical publications were not considered for this review. We also excluded reviews, meta-analyses, guidelines, consensus statements, abstracts, editorials, non peer-reviewed studies, qualitative research, mixed-methods studies, retrospective cohort studies and policy-papers.

### Information sources

A comprehensive search was conducted on 3rd February 2025 in MEDLINE (via PubMed), CINAHL (via EBSCOhost), Cochrane (via Ovid), and Embase (via Ovid) from 1 January 2010 to 31 January 2025. Grey literature was identified on Google Scholar (first 10 pages), and additional publications were identified by reference tracking of included studies. The PROSPERO database was also screened for relevant systematic reviews. Reference lists of included articles and relevant systematic reviews were also screened.

### Search strategy

Search strategies combined controlled vocabulary terms like MeSH headings and free-text terms covering geriatric patients, GR, outcomes related to process and participation (controlled vocabulary terms combined with keywords). The full strategy is provided in Supplementary File 1.

### Selection process

All records were imported into Covidence™ for deduplication and screening. Five reviewers independently screened titles/abstracts (MS, PB, CP, SG, SLR), and full-texts were reviewed by two researchers (MS, CP). Disagreements in title/abstract screening and full text review were resolved by an independent reviewer (MH). Reasons for exclusion were documented for the PRISMA flow diagram.

### Data extraction

Data were extracted using a Covidence™ sheet, with a customised structure based on the defined criteria. Two researchers (MS, CP) independently mapped each study against predefined core components of the GR process according to the primary and secondary outcomes [20, 21]. Extraction discrepancies were resolved by a third researcher (MH).

### Risk of bias assessment

Risk of bias was evaluated using revised JBI critical appraisal tool for RCTs [22], and the JBI checklist for cohort studies, with each item rated as yes/no/unclear/not applicable [23]. The assessment was conducted independently by two researchers (RRW, SLR). Disagreements were resolved by consensus; if necessary, a third person (BN) was consulted. Overall risk of bias was categorised as low, moderate, or high based on a structured qualitative judgement considering both the proportion of fulfilled checklist criteria and the presence of critical methodological limitations. Due to differences in checklist structure across study designs (including sub-items), no strict numerical cut-off values were applied.

### Synthesis methods

Given heterogeneity in context/intervention designs and outcomes, a narrative synthesis was performed. Studies were grouped according to the European Framework of Rehabilitation Services Types [24] (Table 2) to delineate the various stages of the rehabilitation process. Reported process components were mapped, categorised and synthesised across settings. Patterns were compared to identify shared and context-specific elements. Publications arising from the same study were identified, linked, and synthesised at the study level, considering the distinct outcomes. No meta-analysis was conducted.

This review was reported in accordance with the PRISMA 2020 checklist, provided in Supplementary File 2.

## Results

### Study selection

**Fig. 1:**
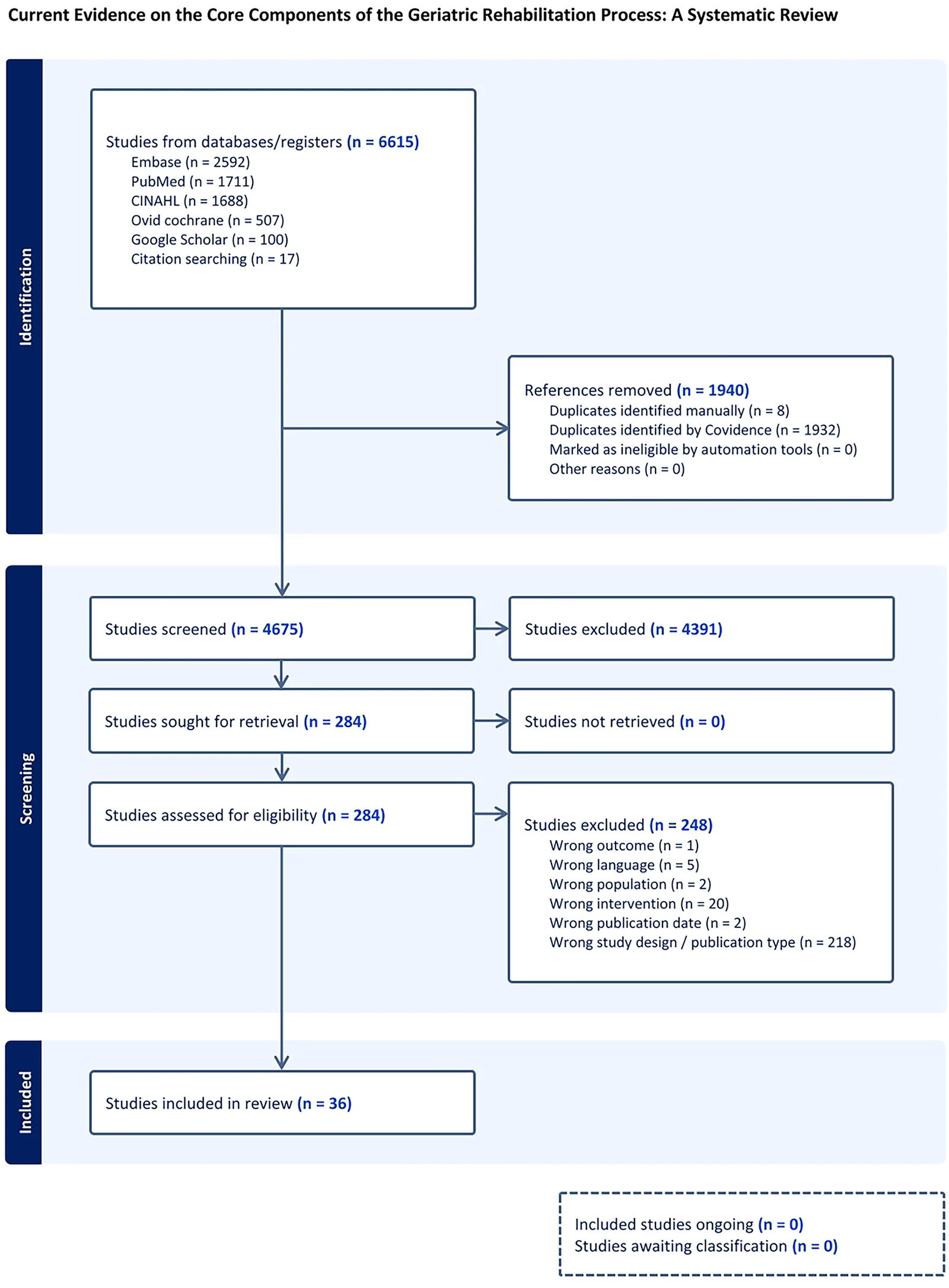
PRISMA flow diagram shows the selection of articles for inclusion in the systematic review

The database search yielded a total of 6615 records, including 117 records identified through manual searching of reference lists and citation tracking. After removal of 1940 duplicates, 4675 records were screened by title and abstract, of which 4391 were excluded as clearly not meeting the inclusion criteria. A total of 284 full-text articles were assessed for eligibility. Of these, 36 studies met the inclusion criteria and were included in the final synthesis. Figure 1 summarises the study identification and selection process.

A full list of excluded studies with reasons is provided in Supplementary File 3.

### Study characteristics

The 36 included studies comprised 31 RCTs and 5 further controlled prospective primary cohort studies with comparison design across 11 countries (Australia = 10, the Netherlands = 5, United Kingdom = 4, Denmark = 3, Taiwan = 3, United States of America = 3, Italy = 2, Japan = 2, Sweden = 2, Canada = 1 and Ireland = 1) [25–60], with the largest number of studies being published between 2018 and 2021.

The present review comprised a total of 10,647 older persons, sample sizes ranged from 32 [41] to 3606 participants [39], with mean ages between 76 [35] and 88.6 years [31].

Included studies covered a range of GR settings according to the European Framework of Rehabilitation Services Types [24] with rehabilitation in acute care [26, 57, 58], followed by rehabilitation in acute care combined with rehabilitation services at home [30], post-acute rehabilitation [27, 32, 33, 39, 40, 42, 43, 49–52, 54, 60], post-acute rehabilitation combined with rehabilitation services at home [28, 37, 41, 47, 55, 56], day hospital/outpatient rehabilitation [29, 45, 46, 59] and rehabilitation services at home [25, 31, 34–36, 38, 44, 48, 53]. Patient characteristics including age, number of participants and population description, are summarised in Table 1.

**Table 1:** Patient characteristics

Study ID	Country	mean *(median)* age (SD)	Number of participants	Population description
[25]	Italy	79 ( ±}6.6) / IG: 77.9 ( ±} 6.0) CG: 79.3 ( ±}7.0)	n = 283 (randomised) n = 245 (6 months follow-up) IG: n = 122 CG: n = 123. Drop-out: n = 38. Deaths: n = 7	Older multimorbid patients (medium/high fall risk, MMSE > 18/19). Female: 59%
[26]	Denmark	78 (72–85) / groupII: 77 (72–84) groupIII: 76 (73–84) groupIV: 80 (72–86) CG: 78 (71–84)	n = 336 (randomised) / n = 272 (analysed) G1: n = 68 G2: n = 70 G3: n = 71 G4: n = 63. Drop-out: n = 64. Deaths: n = 14	Urban/rural nonsurgical older adults (≤ 8 in the 30 s-CST). Female: 61%
[27]	Ireland	82.9 *(median)* / IG: n = 83.1 (6.1), CG: n = 82.7 (6.3)	n = 33 (randomised) n = 27 (analysed) IG: n = 14 CG: n = 13. Drop-out: n = 6. Deaths: n = 5	Heterogenious mildly frail older adults (multiple comorbidities). Equal male/female
[28]	Australia	DG: 78.7 ( ±}8.0), centre group: 79.2 ( ±}7.4)	n = 107 (randomised) n = 76 (completed) DG: n = 41. CG: n = 35. Drop-out: n = 31. Deaths: n = 3	Community-dwelling multimorbid older adults. Female: 66%
[29]	United Kingdom	79 / IG: 79.1 (5.7). CG: 78.4 (5.6)	n = 364 (randomised). n = 344 (analysed primary outcome) (IG: n = 172 CG: n = 172). n = 277 (analysed secondary outcome) (IG: n = 137 CG: n = 140). Drop-out: n = 87, Deaths: 18	Older people at high risk of fall living at home, rural/urban. Female: IG: 58% CG: 62%
[30]	Australia	78 (65–95)	n = 128 (randomised) IG: n = 64 CG: n = 64. n = 107 (6 months) IG: n = 49 CG: n = 58. Drop-out: n = 21. Deaths: notspecified	Multimorbid older patients (risk for readmission). Female: 62%
[31]	Australia	88.6 (5.6) / IG: 88.6 (5.4) CG: 88.6 (5.7)	n = 240 (randomised) n = 126 (12 months follow-up) IG: n = 60. CG: n = 66). Drop-out: n = 114. Deaths: not specified	Hospitalised older adults (recent hip fracture, prior NCF/ambulant, cognitive impairment, ADL dependency). Female (baseline): IG: 73.1% CG: 75.2%
[32]	United States of America	79.3 (8.0)	n = 229 (randomised) IG: n = 114 (E-MR) CG: n = 115	No formal drop-out Older adults in SNFs. Female: 65.1%
[60]*	The Netherlands	IG: 80.7 (SD 6.9) CG: 79.6 (SD 7.1)	n = 149 (baseline). 9 month follow-up: n = 87 (IG: n = 19 CG: n = 68). Drop-out: n = 62. Deaths: n = 30	Community-dwelling multimorbid older adults (complex health problems, prior hospitalised). Female: IG: 67.9% CG: 65% & informal caregivers (if available)
[33]*	The Netherlands	IG: 80.7 (SD 6.9) CG: 79.6 (SD 7.1)	n = 149 (baseline). 9 month follow-up: n = 87 (IG: n = 19 CG: n = 68). Drop-out: n = 62. Deaths: n = 30	Community-dwelling multimorbid older adults (complex health problems, prior hospitalised). Female: IG: 67.9% CG: 65% & informal caregivers (if available)
[34]	Australia	83.3 (5.9) / IG: 83.4 (5.81) CG: 83.2 (5.91)	n = 241 (randomized). Completed 12 month n = 216 (IG n = 107 CG n = 109). Drop-out: n = 25. Deaths: n = 22	Community-dwelling multimorbid frail older adults, MMSE > 18, estimated life expectancy > 12 months). Female: 68%
[35]**	United Kingdom	76 (65–91)	n = 60 (randomised) high intensity IG1: n = 20. moderate intesity IG2: n = 19. standard CG: n = 21 / n = 49 (12 months). IG1 n = 18. IG2: n = 15. CG: n = 16. Drop-out: n = 11. Deaths: 0. (n = 54 carer)	Older patients (mild dementia/cognitive impairment—MoCA 15–25 /MMSE 18–26 or ACEIII 60–94, able to walk and communicate). Female: 43%
[36]	Denmark	88 (range not provided)	n = 238 (tailor-made hospital-at-home: n = 153 standardised home-based rehabilitation: n = 85). Drop-out: n = 52 (90 day mortality)	Older multimorbid frail nursing home residents (hip fracture, > 2/3 dementia, most physically dependent). Female: Tailor-made: 83%, standardised: 68%
[37]***	Australia	82.7 / IG: 82.4 ( ±} 5.7) CG: 83.0 ( ±} 6.2)	n = 175 (randomised). n = 134 (analysed at 12 months) IG: n = 68 CG: n = 66. Drop-out: n = 41. Deaths: n = 19	Community-dwelling older adults (proximal femoral fracture, with MMSE ≥ 18. BMI 18.5–35). Female (randomised): 77.1%, IG: 67.4% CG: 86.5%
[38]**	United Kingdom	80 ( 65–95) *(median)* / IG: 80 (75–85) CG: 81 (75–84)	n = 365 (randomised) n = 290 (analysed) IG n = 149 CG n = 141. Drop-out: n = 75. Deaths: n = 11	Older multimorbid adults (early dementia/MCI). Female (randomised): IG: 45% CG: 40%
[39]	Australia	IG: 81.4 (9.3) CG: 82.1 (8.3)	n = 3606 / IG: n = 1623 CG: n = 1983. Drop-out: n = 41 (during admission). Deaths: n = 41	Older multimorbid patients (MMSE > 23 or AMTS > 7/10), complex conditions). Female: IG: 62% CG: 61%
[40]	The Netherlands	80 (72–85) *(median)* / Baseline cohort: 80 (71–85) / follow-up cohort: 79 (71–84)	n = 743 (baseline cohort: n = 386 follow-up cohort: n = 357). n = 637 (analysed) (baseline cohort: n = 331 follow-up cohort: n = 306). Drop-out: n = 106. Deaths: n = 43	Older persons in SNF and & care professionals. Female: 64.5%
[41]	Italy	79.03 ( ±} 7.8) / IG: 80.33 ( ±} 6.72) CG: 77.65 ( ±} 8.40)	n = 32 (randomised) n = 31 (analysed) IG: n = 16 CG: n = 15. Drop-out: n = 1. Deaths: n = 0	Older osteoporotic patients (3 months after hip fracture). Female: 84%
[42]	Taiwan	83.4 ( ±}5.5) / IG: 84.0 ( ±}5.5) CG: 83.1 ( ±}5.5)	n = 541 (allocated) n = 458 (analysed) IG: n = 159 CG: n = 299. Drop-out: n = 83. Deaths: n = 0	Older people (functional decline after acute illness). 100% male
[43]	United States of America	79.3 (8.0) / IG: 79.5 (8.2) CG: 79.2 (7.7)	n = 229 (randomised). n = 186 (completed day 90) IG: n = 90 CG: n = 96. Drop-out: n = 43. Deaths: 17	Older adults (from acute care hospital). Female: 65.1%
[44]	United States of America	80.8 / IG: 80.3 (8.0) CG: 81.2 (8.8)	n = 210 (randomised) n = 197 (analysed) IG: n = 96 CG: n = 101. Drop-out: n = 13. Deaths n = 2	Community-dwelling older patients (hip fracture surgery). Female: 76.7%
[45]	Japan	IG: 83.1 (7.7) CG: 81.8 (8.4)	n = 67 (randomised) n = 63 (analysed) IG: n = 31 CG: n = 32. Drop-out: n = 4. Deaths: n = 0	Community-dwelling frail older patients (musculoskeletal disorders without COPD, MMSE > 21). Female (randomised): IG: 68% CG: 72%
[60]	Japan	IG: 81.5 (73.2–87.2) CG: 79.0 (68.5–80)	n = 35 (randomised) n = 30 (analysed) IG: n = 15 CG: n = 15. Drop-out: n = 5. Deaths: 0	Community-dwelling frail elderly people (chronic stroke). Female: IG: 53% CG: 39%
[47]***	Australia	IG: 82.4 (5.7) CG: 83.0 (6.2)	n = 175 (randomised) n = 165 (analysed). Drop-out: n = 10.Deaths: n = 8	Community-dwelling older adults (fall-related hip fracture, MMSE ≥ 18, BMI 18.5–35). Female (randomised): IG: 67.4% CG: 86.5%
[48]	Canada	77.3 (6.5) / IG: 78.2 (7.0) CG: 76.4 (5.8)	n = 70 (randomised) n = 67 (baseline assessment) IG: n = 35 CG: n = 32. Deaths: n = 1	Community-dwelling older adults (CFS 4–6, independent with/ without walking aid). Female: 77.6%
[49]	United Kingdom	mean 84.1 (6.3) / IG: 84.5 (5.9) CG: 83.6 (6.6)	n = 250 (randomised) IG: n = 106 CG: n = 106 (analysed). Drop-out: n = 38. Deaths: n = 12	Frail multimorbid older people. Female: 64%
[51]****	Australia	IG: 81 (77–88) CG: 81 (77–87)	n = 198 (randomised) n = 157 (analysed) IG: n = 79 CG: n = 78. Drop-out: n = 41	Multimorbid older adults (goal to improve walking /mobility). Female: IG: 60% CG: 55%
[50]****	Australia	80.9 *(median)* (IQR 76.6–87.2)	n = 198 (randomised) IG: 99 CG: 99 / n = 194 (analysed) responder: n = 130 non-responder: n = 64. Drop-out: n = 4. Deaths n = 21	Multimorbid older adults (goal to improve walking/mobility). Male: responders 42% non-responders: 45%
[52]	The Netherlands	82.5 (7.6) / IG: 83.7 (7.3) CG: 81.3 (7.9)	n = 77 (baseline) n = 68 (discharge) IG: n = 34 CG: n = 34. n = 50 (6 months follow-up) IG: n = 24 CG: n = 26. Drop-out: n = 27. Deaths: n = 5	Multimorbid older patients (recent hip-fracture, concerns to fall). Female: 79.2%
[53]	Taiwan	Comprehensive care group: 76.46 (7.14), interdisciplinary care group: 76.17 (6.65) CG: 76.91 (8.20)	n = 299 (randomised) n = 269 (1 year follow up) comprehensive care group: n = 92, interdisciplinary care group n = 92, CG: n = 85. Drop-out: n = 30. Deaths: n = 28	Older patients (hip fracture). Female: comprehensive care group: 59.6% interdisciplinary care group: 67.3% CG: 64.4%
[54]	Australia	IG: 80 (8) CG: 81 (7)	n = 100 (randomised) n = 64 (analysed) IG: n = 29 CG n = 35. Drop-out: n = 36. Deaths: n = 3	Deconditioned older adults (following acute medical condition, prior independently living). Male: IG: 28% CG: 25%
[55]	Taiwan	78.12 (8.08) / IG: 78.61 (8.04) CG: 77.42 (8.38)	n = 176 (randomised) n = 111 (24 months follow-up) IG: n = 53 CG: n = 58. Drop-out: n = 65. Deaths: n = 27	Older adults (diabetes mellitus, hip fracture surgery). Female: 77.3%
[56]	The Netherlands	IG: 78.9 (7.0) CG: 79.0 (6.5)	n = 190 (randomised) n = 143 (completed) IG: n = 77 CG: n = 66. Drop-out: n = 47. Informal caregivers n = 172. Deaths: n = 14	Community dwelling older patients (after stroke). Female: 60.5%. & informal caregives
[57]	Sweden	IG: 87.5 (75–101) CG: 86.2 (76–98)	n = 155 (baseline/analysed) IG: n = 78 CG: n = 77. n = 94 (6 months follow up) IG: n = 46 CG: n = 48. Drop-out: n = 61. Deaths: n = 46	Frail older people (acute hospital admission). Female: IG: 60.3% CG: 55.8%
[58]	Sweden	IG: 87.5 (75–101) CG: 86.2 (76 -98)	n = 178 (randomised) n = 155 (analysed intention to treat) IG: n = 78 CG: n = 77. n = 78 (analysed complete cases) IG: n = 38 CG: n = 40. Drop-out: n = 85. Deaths: n = 67	Frail older people (some with cognitive impairments). Female: IG: 47% CG: 43%
[59]	Denmark	79 ( ±} 8.1) / IG: 78.8 ( ±} 8.3) CG: 78.4 ( ±} 7.9)	n = 368 (randomised) IG: n = 185 CG: n = 183. No drop-outs	Multimorbid older persons. Female: 51%

^*^ Studies from the same underlying programme.

SD: Standard deviation, IQR: Interquartile range, IG: Intervention group, CG: Comparison group, DG: Domiciliary group, NCF: Nursing care facility, MMSE: Mini-Mental State Examination, 30 s-CST: 30 Second Chair Stand Test, ADL: Activities of Daily Living, SNF: Skilled Nursing Facility, E-MR: Enhanced Medical Rehabilitation, COPD: Chronic Obstructive Pulmonary Disease, BMI: Body Mass Index, MoCA: Montreal Cognitive Assessment, ACEIII: Addenbrooke’s Cognitive Examination, CFS: Clinical Frailty Scale, AMTS: Abbreviated Mental Test Score, MCI: Mild cognitive impairment.

### Risk of bias assessment

Risk of bias varied across studies. Among RCTs, 18 were judged as low risk [25, 26, 28–32, 34, 37, 39, 43, 46–48, 52, 54, 56, 59], 13 as some concerns [27, 35, 38, 41, 44, 45, 49–51, 53, 55, 57, 58] according to the revised JBI critical appraisal tool for RCTs [22]. Common issues included inadequate reporting of allocation concealment, lack of blinding of outcome assessment, and incomplete outcome data.

Two of the further five controlled prospective primary cohort studies were judged as moderate risk of bias [36, 40], particularly in confounding control and participant selection (given by the nature of the methodology). A detailed assessment for each study is provided in Supplementary File 4.

### Results of individual studies

The conceptualisations of the GR process presented heterogeneity across the studies. Table 2 includes an overview of intervention details and process related findings of the studies. The functional assessments and outcome parameters used varied: Most frequently used were Mini-Mental State Examination (MMSE) [25, 31, 34, 35, 37, 39, 42, 44, 46, 47, 49–53, 57–59], Barthel Index (BI) [25, 26, 29, 31, 32, 34, 36, 40, 42, 43, 46, 49, 53, 55, 59], and other assessments of Activity of Daily Living (ADL) (Instrumental ADL—IADL, Personal ADL-PADL, Nottingham Extended ADL-NEADL) [29, 30, 34, 35, 37, 38, 55, 57, 58], European Quality of Life 5 Dimensions (EQ-5D) [25–28, 33, 38, 49, 51], Timed Up and Go Test (TUG) [25, 27, 28, 30, 35, 38, 41, 51], Hand Grip Strength [27, 37, 38, 41, 45, 46, 57, 58], Berg Balance Scale (BBS) [25, 30, 35, 38, 40, 57, 58], Falls Efficacy Scale-International (FES-I) [25, 29, 31, 35, 38, 52], Charlson Comorbidity Index (CCI) [36, 49–51, 54, 59], 6-Minute Walk Test (6MWT) [43–46, 50, 51], as well as Body Mass Index (BMI) [28, 37, 42, 44, 47], Gait Speed [34, 37, 43, 57, 58] and Geriatric Depression Scale (GDS) [42, 50–53]. CGA as such was used in 7 studies [31, 34, 42, 56–59].

**Table 2:** Design, intervention, process related findings, risk of bias

**Study ID**	**Study design**	**Intervention type, rehabilitation setting, duration**	**Professionals responsible for the intervention**	**Comparison**	**Used assessments**	**Process related findings (message)**	**RoB**	***European Rehabilitation Service types (mapping)***
[25]	RCT	Telerehabilitation to prevent falls, home-setting, 6 months follow-up.	Physician, PT, nurse	Usual care (GP, monthly falls check per phone)	BI, BBS, EQ-5D; FES-I, MMSE, TUG, CIRS Severity & Comorbidities Index	Feasible, safe, effective	low risk	Rehabilitation services at home
[26]	RCT (two-way factorial)	Systematic functional assessment and immediate rehabilitation (4 groups), inpatient setting, median LOS 4, follow up median 23 days	Physician, PT, OT, nurse	Usual assessment, usual rehabilitation (group 1)	BI, EQ-5D, 30s-CST	No significant difference in physical performance	low risk	Rehabilitation in acute care
[27]	RCT (phase 1 prospective single-blinded feasiblity)	PRT, inpatient setting, 6 weeks.	PT	Usual care (PT 5x/week)	EQ-5D, Hand Grip Strength, TUG, VAS SNT, LLstrength, CFS	PRT is safe and acceptable, effectiveness not demonstrated	moderate risk	Post-acute rehabilitation
[28]	RCT (blinded baseline assessment, 8 weeks, 6 months follow-up)	Multicomponent falls prevention rehabilitation (ProFaNE), home-based vs. centre-based setting, 8 weeks / 6 months follow up.	Physician, PT, OT	n.a.	EQ-5D, TUG, 9-Hole Peg Test, ONI, Kessler psychological distress K10-scale, AMTS, Romberg, FAI, BMI, Quads strenth (kg), Lateral pinch test (kg), Step Test, Simple reaction test (ms), Caregiver strain, Nutrition score	Provision of centre-based programs for communitydwelling older adults with falls history advised	low risk	Post-acute rehabilitation Rehabilitation service at home
[29]	RCT (multi-centre)	Multifactorial falls prevention program, geriatric day hospital setting, 6+ sessions.	Physician, PT, OT, nurse	Usual care	BI, FES-I, NEADL	No conclusively benefit of day-hospital multifactorial falls prevetion programme	low risk	Day rehabilitation
[30]	RCT	Individually tailored programme for exercise and follow-up care, hospital, home transitional care model, visits and phone calls, 24 weeks	PT, nurse	Usual care	BBS, TUG, IADL, WIQ	Transitional care model can improve independence and functional ability	low risk	Rehabilitation in acute care Rehabilitation services at home
[31]	RCT (parallel trial)	4-week multidisciplinary program after hip-fracture & health economic study, outreach home setting (NCFs), 4 weeks, 12 months follow-up.	Physician, PT	Usual care	BI (modified), CGA, DEMQOL, EQ-5D, MMSE, NHLSD, PAINAD, Cornell Scale for Depression, Functional Recovery Scale, Confusion Assessment Method, MNA, QALYs	Outreach rehabilitation improved mobility, nutritional status and survival at 4 weeks. No persistance at 12 months. Not cost-effective.	low risk	Rehabilitation services at home
[32]	RCT (secondary analysis)	Enhanced Medical Rehabilitation (E-MR), inpatient setting (SNF), LOS (d): 23.5 (13.1) (standard duration).	PT, OT, Certified therapy assistant	Usual care (standard rehabilitaiton)	BI, Clock drawing test, SBT, MADRS PANAS, CIRS-G, Readiness for Rehab, PRPS	EM-R achieved greater functional improvement.	low risk	Post-acute rehabilitation
[60]*	Prospective cohort study	Effectiveness of an integrated GR care pathway, hospital - GR - primary aftercare at home, 9 months.	Physician, PT, OT, care pathway coordinator	Usual care	Katz-15, FAI, IPA, RAND-36, CSAL, SRB-VAS, Erasmus iBMG	Recommendation to implement the care pathway in regular care.	low risk	Post-acute rehabilitation
[33]*	Prospective cohort study (economic evaluation)	Economic evaluation of an integrated GR care pathway, hospital - GR -primary aftercare at home, 9 months.	Physician, PT, OT, care pathway coordinator	Usual care	EQ-5D, KATZ-15, QALY	Integrated care pathway resulted in less dependence in ADL and fewer costs.	low risk	Post-acute rehabilitation
[34]	RCT (parallel-group assessorblind, single centre)	Multifactorial interdisciplinary intervention on mobility related disability, home-setting, 12 months.	Physician, PT, OT, nurse, dietician	Usual care (GP & community health services)	BI, GAS, MMSE, SPPB, AMPAC, Reintegration to Normal Living Index, Life Space Assessment, NEADL, Gait speed Satisfaction with outdoor mobility	Multifactorial interdisciplinary program targeting frailty was more effective in reducing mobility-related disability at the participation and activity levels	low risk	Rehabilitation services at home
[35]**	RCT (multi-site feasibility)	PrAISED: Test of 2 versions - Promoting activity Independence and stability in early dementia (high and moderate intensity), home-setting, 12 months.	Physician, PT, OT, Rehabilitation support Worker	Usual care	BBS, DEMQOL, EQ-5D, FES-I, HADS, TUG, DAD, NEADL, IPAQ Physical Activity Score, SHARE Frailty Index, CANTAB, CSI, ACEIII, MMSE, MoCA	A larger trial is feasible with adjustments	moderate risk	Rehabilitation services at home
[36]	Prospective cohort study	2 new geriatric multidisciplinary intervention approaches (tailor-made hospital-at-home & standardised home-based rehabilitation) after discharge from orthopedic surgery (hipfracture), home-setting, duration app. 30 days after discharge.	Physician, PT, nurse	Usual care (standardised rehabilitation)	BI (modified), CCI	Hospital-at-home immediately after discharge by multidisciplinary team appeared to have a positive impact on short-term mortality and acute readmission.	moderate risk	Rehabilitation services at home
[37]***	RCT (four site, blinded assessed outcomes, 12 month follow-up)	INTERACTIVE: Individual combined exercise-and-nutrition therapy, inpatient setting (post hip fracture surgery), continued after discharge to community, 6 months.	PT, dietician	Usual care	Hand Grip Strength, MMSE, BMI, Gait speed, Knee strength with NMMT, MoBERG, PADL, IADL, AQol	Individual nutrition therapy and exercise did not significantly improve gait speed compared with standard care.	low risk	Post-acute rehabilitation Rehabilitation service at home
[38]**	RCT (multi-centre individual randomised stratified pragragmatic parallel group)	PrAISED: individually tailored home rehabilitation programme - Promoting Activity Independence and Stability in Early Dementia and mild cognitive impairment specially designed dementia specific rehabilitation programme, home-setting, 12 months.	Physician, PT, OT, Rehabilitation supportWorkers	Usual care	BBS, DEMQOL, EQ-5D, FES-I, HADS, Hand Grip Strength, MoCA, TUG, (dual task) TUG, NEADL, DAD, LAPAQ, CANTAB, apathy evaluation scale, SHARE frailty instrument, Accelerometer, Verbal fuency-correct words, Client service receipt inventory, CSI	No improvement in ADL, physical activity, QoL, falls rate or other health status outcomes.	moderate risk	Rehabilitation services at home
[39]	RCT (cluster steppedwedge, multi-centre)	Individualised patient education on falls prevention + staff training and feedback, inpatient setting (hospital ward unit), 50 weeks.	PT, nurse, allied health Staff	Usual care (hospital-wide falls prevention program, nurseled education, environmental adaptations)	FIM, MMSE, AMTS	Reduced falls and injurious falls through individualised patient and staff education during rehabilitation	low risk	Post-acute rehabilitation
[40]	Prospective longitudinal study, multi-centre (2 consecutive cohorts, 1 year follow-up) – SINGER	National programme to stimulate self-organising capacity to develop integrated care to improve GR in 4 domains of health service delivery (SINGER), inpatient setting, 1 yearpost implementation.	Physician, PT, nurse	n.a. - 2 consecutive chorts	BI, BBS, FCI, CPS, DRS, FAC	More independence in ADL at discharge 1 year post implementation. Combined outcome (independence in ADL /discharge home / short LOS) only significantly improved in traumatic injury patients.	moderate risk	Post-acute rehabilitation
[41]	RCT (pilot)	Supplemention of amino acid during rehabilitation on functioning in hip fracture patients, outpatient & homesetting, 8 weeks.	PT, dietician	Usual care (physical exercise rehabilitation and dietetic counseling)	Hand Grip Strength, TUG, SPMSQ, CIRS, SMI, BIA, ILOA, HRQoL, SF-12, MCS, PCS, ASMMI	Multidisciplinary rehabilitative and nutritional intervention seems effective on functioning in hip fracture patients (particular sarcopenic ones).	moderate risk	Post-acute rehabilitation Rehabilitation service at home
[42]	Cohort study	Doubling the dose of physical therapy, inpatient setting (post-acute care unit), 4 weeks.	PT	Usual care (usual rehabilitation group)	BI, CGA, GDS, MMSE, BMI, Subjective pain perception, NRS	Intensified rehabilitation significantly improved physical function / depressive symptoms and pain.	low risk	Post-acute rehabilitation
[43]	RCT (double-blind parallel group)	Effect of E-MR (PT, OT) on functional recovery, inpatient setting (SNF), LOS: 23.5 (13.1) - timepoints: 30/60/90 days post discharge.	PT, OT	Usual care (rehabilitation (PT, OT without E-MR)	BI, 6MWT, Gait Speed, Montgomery-Asberg Depression Rating Scale, Short Blessed Test Score, Cumulative Illness Rating Scale for Geriatrics, PRPS	E-MR is effective in improving functional recovery.	low risk	Post-acute rehabilitation
[44]	RCT (parallel 2 group, multicentre)	Multicomponent home-based physical therapy intervention on ambulation after hip fracture, home-setting, 16 weeks.	PT, dietician	Usual care (additional transcutaneous electrical nerve stimulation, active range-of-motion exercises)	6MWT, SPPB, CES-D, MNA-SF, BMI, 3MS, modified Physical Performance Test, NHATS balance score	No effects.	low risk	Rehabilitation services at home
[45]	RCT (open-label)	Additional respiratory (to typical) rehabilitation, day rehabilitation setting, 12 sessions twice a week for 6 weeks.	PT	Usual care (typical rehabilitation)	FIM, Hand Grip Strength, 6MWT, SF-8, DRACE, RSST	Improvement of respiratory function / swallowing and QoL by the addition of respiratory rehabilitation suggested.	moderate risk	Day rehabilitation
[46]	RCT (open)	Additional pulmonary training and home exercise program, day rehabilitation setting, 12 sessions twice a week for 6 weeks.	Physician, PT	Usual care (standard rehabilitation)	BI, Hand Grip Strength, MMSE, 6MWT, DRACE, MOS-SF8	Improvement of pulmonary function / deglutition and QoL by the addition of pulmonary training including home exercise regimens suggested.	low risk	Day rehabilitation
[47]***	RCT (blinded outcome assessment) – costeffectiveness	INTERACTIVE: (individual combined exercise-and-nutrition therapy): Costs for utilisation of hospitals / health and community services compared with quality-adjusted life years gained (cost-effectiveness), 6 months.	PT, dietician	Usual care (rehabilitation programme)	MMSE, AQoL, ICER, BMI, QALY	Indication of cost-effectiveness (high level of uncertainty).	low risk	Post-acute rehabilitation Rehabilitation service at home
[48]	RCT (parallel group, feasibility)	Virtual frailty rehabilitation program (Geras) to build resilience in older adults with frailty during COVID-19 comprising of exercise, medication support, nutrition and socialisation, home-setting, 12 weeks.	PT, research assistant, pharmacist, social call volunteers	Sozialisation arm (weekly phone call)	5XSST, DASS-21	Virtual rehabilitation feasible, (no significant changes in clinical outcomes between arms)	low risk	Rehabilitation services at home
[49]	RCT (single-centre pragmatic with health economic study)	Comparison of community In-reach Rehabilitation and Care Transition (CIRACT) service and traditional hospital-based rehabilitation (THB-Rehab) service, inpatient & home setting, LOS: median 8 vs. 9 days.	Physician, PT, OT	Usual care (traditional hospital rehabilitation)	BI, CCI, EQ-5D, MMSE, QALYs, CSRI	No reduction in hospital LOS, readmission reates of CIRACT, cost-effectiveness uncertain.	moderate risk	Post-acute rehabilitation
[51]****	RCT (multi-centre, parallelgroup, Concealed allocation, assessor/investigator blind, intention-to-treat analysis)	Multidisciplinary rehabiltation with additional physical activities emphasising mobility, inpatient setting, additional 1-2 sessions per day, LOS 20.2, 6 months follow-up.	PT, OT, nurse	Usual rehabilitation plus equal time in social activities	CCI, EQ-5D, FIM, GDS, MMSE, 6MWT, TUG, DEMMI, Fried Frailty Scale	No faster self-selected gait speed at discharge or improvements in other outcomes.	moderate risk	Post-acute rehabilitation
[50]****	RCT (investigator/assessor blinded parallel group, multi-centre)	Secondary analysis (whole group). (setting duration see above).	PT, assistant	n.a.	CCI, GDS, MMSE, Fried Frailty Scale, 6MWT	Increased days of rehabilitation and better cognition (MMSE) were linked to higher odds of a positive rehabilitation response.	moderate risk	Post-acute rehabilitation
[52]	RCT (cluster)	Effects of FIT-HIP (multicomponent cognitive behavioral) intervention for fear of falling after hip fracture, inpatient setting, duration: LOS median 38 d.	PT, nurse, psychologist	Usual care (multidisciplinary inpatient GR)	FES-I, GDS, HADS, MMSE, POMA, FAC, 10-Meter Walk Test, FCI	No effectiveness in reducing FoF.	low risk	Post-acute rehabilitation
[53]	RCT (experimental, 3 models)	Comparison of a comprehensive care program with interdisciplinary and ususal care after hip fracture (ICG: acute/subacute and transitional interventions, CCG additional nutrition consultation / depression management and fall prevention), home-setting, ICG 4 months, CCG 12 months (both 3 PT sessions in 4 months, CCG additional session in 6th month).	Physician, PT, nurse, dietician	Usual care (in-hospital rehabilitation)	GDS, Chinese BI, MNA, CMMSE	More benefits from comprehensive care.	moderate risk	Rehabilitation services at home
[54]	RCT (single-blind)	OT and individual activity pacing (5x), activity pacing education, activity pacing review session, inpatient setting, LOS 14 days.	OT	Usual care	CCI, FIM, AusTOMs-OT, SF-36, SEG, APA	Activity pacing in addition to typical OT did not demonstrate benefits in management of their daily activities.	low risk	Post-acute rehabilitation
[55]	RCT (effectiveness evaluation, experimental design)	Diabetes specific care model for hip fractured patients - interdisciplinary care component including geriatric consultation, inpatient (hospital) and home-setting, duration: 4 months after discharge, assessments 1,3,5,12,18 and 24 months (follow up).	Physician, PT, nurse	Usual care (PT and nursing care, discharge 4-5 days after sugery)	BI, ADL/IADL	Enhancement of physical functions of the affected limb during 24 months following discharge. No significant effects in improving self-care ability/mortality/subsequent falls/hospital readmissions/emergency department visits and institutionalisation rate.	moderate risk	Post-acute rehabilitation Rehabilitation services at home
[56]	RCT (two-arm, multicentre)	Integrated multidisciplinary GR after stroke (3 modules: inpatient neurorehabilitation, home-based selfmanagement, education for patients and informal caregivers), inpatient and home-setting, 2-6 months.	Physician, PT, OT, (neuro)psychologist, speech therapist, stroke care coordinator	Usual care (multidisciplinary inpatient neurorehabilitation without coordinated aftercare)	CGA, KATZ-15, FAI, SSQol, Self-Rated Burden VAS scale, IPA, Carer Quality of Life, Erasmus iBMG	No effect on daily activity. Higher level of perceived autonomy of outdoor activities, lower perceived care burden for informal caregivers.	low risk	Post-acute rehabilitation Rehabilitation services at home
[57]	RCT (two-arm) - CGA-Swed	CGA by multidisciplinary team for individual tailored rehabilitation, inpatient setting (geriatric acute ward).	Physician, PT, OT, nurse, dietician, social worker	Usual care (acute hospital)	CGA, BBS, Hand Grip Strength, MMSE, PADL, IADL, SF-36, Fugl-Meyer-Lisat-11, Pyramid Questionnaire, Fried Frailty Scale, CIRS-G	No significant results in ADL. (CGA during hospital stay probably not enough for long-term effects)	moderate risk	Rehabilitation in acute care
[58]	RCT (two-arm intervention study) - CGA-Swed	Effects on ADL one year after CGA performend in inpatient setting(hospital), 12 months follow up.	Physician, PT, OT, nurse, dietician, social worker	Usual care (medical ward at hospitals)	CGA, BBS, Hand Grip Strength, MMSE, IADL, PADL, CIRS-G, gait speed	Significant improvement in ADL after 1 year with CGA during hospital stay, increased ability to remain in own housing.	moderate risk	Rehabilitation in acute care
[59]	RCT (open assessorblinded, two parallel group)	Geriatrician-performed omprehensive geriatric care (individual disease management), outpatient setting, 5 weeks. Assessment 10, 30, 90 days post admission.	Physician, PT, OT, nurse, dietician	Usual care (5 weeks rehabilitation)	modified BI, CCI, MMSE, DL, START / STOPP	No effect on secondary healthcare utilisation, maybe reduced primary healthcare utilisation and improved overall quality of life during 90-day follow-up.	low risk	Outpatient rehabilitation

^*^ Studies from the same underlying programme.

GP: general practicioner, PT: physiotherapist, BI: Barthel Index, BBS: Berg Balance Scale, EQ-5D: European Quality of Life 5 Dimensions, FES-I: Falls Efficacy Scale-International Version, MMSE: Mini Mental State Examination, TUG: Timed Up & Go, CIRS(-G): Cumulative Illness Rating Scale(-Geriatric), LOS: length of stay, OT: occupational therapist, 30 s-CST: 30 Second Chair Stand Test, PRT: progressive resistance training, VAS: Visual Analogue Scale, LL: lower limb, CFS: Clinical Frailty Scale, ONI: Ongoing Needs Identification, AMTS: Abbreviated Mental Test Score, FAI: Frenchay Activities Index, BMI: Body Mass Index, CSI: Caregiver Strain Index, ADL: Activities of Daily Living, CGA: Comprehensive Geriatric Assessment, IADL: Instrumental Activities of Daily Living, PADL: Personal Activities of Daily Living, NEADL: Nottingham Extended Activities of Daily Living, WIQ: Walking Impairment Questionnaire, DEMQOL: Dementia Quality of Life Instrument, NHLSD: Nursing Home Life-Space Diameter, PAINAD: Pain Assessment in Advanced Dementia, MNA(-SF): Mini Nutritional Assessment (-Short Form), QALYs: Quality-Adjusted Life Years, SBT: Short Blessed Test, MADRS: Montgomery Asberg Depression Rating Scale, PANAS: Positive and Negative Affect Schedule, RFR: Resting Full-Cycle Ratio, PRPS: Pittsburgh Rehablitation Participation Scale, IPA: Impact on Participation and Autonomy, SRB-VAS: Self-Related Burden Visual Analogue Scale, GAS: Goal Attainment Scaling, SPPB: Short Physical Performance Battery, AMPAC: Activity Measure for Post Acute Care, HADS: Hospital Anxiety and Depression Scale, DAD: Disability Assessment For Dementia, IPAQ: International Physical Activity Questionnaire, CANTAB: Cambridge Neuropsychological Test Automated Battery, ACEIII Addenbrooke’s Cognitive Examination, MoCA: Montreal Cognitive Assessment, AQol: Assessment of Quality of Life, CPS: Cognitive Performance Scale, DRS: Dementia Rating Scale, SPMSQ: Short Portable Mental Status Questionnaire, SMI: Severe Mental Illness, HRQoL: Health-Related Quality of Life, GDS: Geriatric Depression Scale, 6MWT: 6-Minute Walk Test, FIM: Functional Independence Measure, DRACE: Dysphagia Risk Assessment for the Community-dwelling Elderly, CCI: Charlson Comorbidity Index, SF-8: Short-Form 8, SF-12: Short Form-12, SF-36: Short Form-36, 5XSST: 5X Sit-to-Stand Test, DASS-21: Depression, Anxiety, and Stress Symptoms, DEMMI: de Morton Mobility Index, POMA: Tinetti Performance-Oriented Mobility Assessment, AusTOMs-OT: Australian Therapy Outcome Measures for Occupational Therapy, SSQol: Stroke-Specific Quality of Life Scale, SNT: Stair Negotiation Test, CSAL: Cantril’s Self Anchoring Ladder, KATZ-15: Modified version of the Katz Index of Independence in Activities of Daily Living, NMMT: Nicholas manual muscle tester, MoBERG: modified Berg Balance scale, LAPAQ: Longitudinal Study of Ageing Amsterdam Physical Activity Questionnaire, SHARE: Survey of Health, Ageing, and Retirement in Europe, FCI: Functional Comorbidity Index, FAC: Functional Ambulation Categories, BIA: Bioelectrical impedance analysis, ILOA: Iowa Level of Assistance scale, PCS: Physical Composite Score, MCS: Mental Health Composite Score, ASMMI: appendicular skeletal muscle mass index, NRS: numerical rating scale, CES-D: Center for Epidemiologic Studies–Depression scale, 3MS: modified Mini-Mental State Examination, NHATS: National Health and Aging Trends Study, RSST: Repetitive Saliva a Swallowing Test, MOS-SF8: Medical Outcome Study 8-Item Short-Form Health Survey, ICER: Incremental cost-effectiveness ratio, CSRI: Client Service Receipt Inventory, SEG: Self-Efficacy Gauge, APA: Activity Pacing Assessment, IPA: Impact on Participation and Activity, DL: Depression List, START: Screening Tool to Alert doctors to Right Treatment, STOPP: Screening Tool of Older Person’s Prescriptions.

Studies reveal also variations according to GR settings, interventions and components of the core process. However, GR services at home seem feasible and effective, though not consistently [25, 28, 31, 34–36, 38, 44, 48, 53, 55, 56]. In post-acute rehabilitation, most studies focused on therapeutic interventions, like enhanced medical rehabilitation approaches [32, 43], higher therapy intensity [42, 45, 46], education [39], nutritional interventions [41] and fixed, function-oriented programmes without individualised approaches at the activity and participation level [27, 29, 52, 54]. The use of assessments like CGA seems to have a positive impact on long-term success [57, 58], and cognitive performance also implies an impact on outcomes [50, 51]. An integrated care pathway was also deemed essential for participation in life [30, 40, 60]. Process-related economic perspectives with different approaches were evaluated in four studies [31, 33, 47, 49]. Further details of individual studies are shown in Table 2.

Our narrative synthesis without pooled effect estimates across all included studies showed that GR care process components were largely aligned with the domains described in the European consensus statement by Grund et al. [6]. These included eligibility criteria for GR admission, functional and QoL outcomes, use of assessments, patient-centred and multidisciplinary treatment processes, as well as individualised discharge and care transition management, with additional consideration of the contextual requirements of GR.

Marked heterogeneity existed in terminology and depth of reported process descriptions, team composition, workflow sequencing, terminology, and inclusion of contextual information. A notable difference was also observed in reported relation to the type of intervention, the setting of GR, the duration, the frequency and the more than 90 different assessments conducted. This variability limited comparability across studies.

No study described the entire holistic GR process, starting with admission to acute geriatric rehabilitative care, continuing with CGA-based, individualised post-acute GR based on patient-centred rehabilitation goals, individualised therapy planning, and followed by individualised GR aftercare services for older patients as discussed in previous literature [7, 8]. A total of three studies were able to trace the overall process to a certain extent by describing an integrated care pathway [40, 49, 60]. The interventions encompassed a wide range of approaches with varying contents, including comprehensive, multiprofessional programmes, and fixed therapy regimes delivered across diverse settings and varying team compositions. These results show clearly the heterogeneity, fragmentation, and poorly standardised process reporting.

Considering the domains of the ICF, the majority of studies were function-oriented, with programmes not based on personalised goal-setting according to the biopsychosocial model [12, 61]. Participation and activity were considerably underrepresented. In most studies, no CGA was performed. Moreover, the majority of studies excluded patients with moderate or severe dementia. The multimorbidity of the study participants was also evident from the fact that 1369 of 10,647 patients were unable to complete GR as planned, of whom a total of 516 died during the study period.

## Discussion

This systematic review aims to identify and synthesise existing evidence on the core components of a person-centred GR process. In order to reach these goals authors deliberately restricted inclusion to RCTs and controlled prospective primary cohort studies because the objective was to identify key components of GR care that are not only described as “good practice”, but are supported by comparative evidence for patient-relevant benefit [62, 63].

As the included studies span several geographical regions, the results highlight the relevance of this topic across different populations and healthcare contexts. However, the studies showed heterogeneity in reporting GR process results, including acute, inpatient, outpatient, and geriatric telerehabilitation settings. This fact may have impacted the care process itself, the staffing for GR, therapeutic interventions, but also the use of assessment instruments. The most commonly used assessment tools were the MMSE, the BI and other assessments of ADL demonstrating that physical and mental independence were highly valued. Interestingly, the CGA as such was only stated in 7 studies [31, 34, 42, 56–59] even though this assessment forms the basis for tailored and personalised medicine [64, 65]. Functional recovery is an intended target of GR, but a rehabilitation orientation that is defined solely by functional metrics risks missing activity and participation-related outcomes that older people consistently describe as most important. Older patients prioritise maintaining independence over life extension. ‘Success’ is often framed in terms of autonomy, not isolated performance gains [66]. This highlights the importance of the GR team collaborating with informal caregivers to prioritise facilitating discharge home, with the objective of restoring life participation to the greatest extent possible. In addition, a scoping review of the quality of GR from the patient perspective highlighted needs that extend beyond function, including being heard, receiving information, psychological and social support, and shared decision-making [67]. More broadly, qualitative work on older people’s goals of care showed that goals are frequently articulated as preserving well-being in one’s lifeworld, maintaining dignity and relationships, and adapting meaningful activities in the face of limitations, domains that are not captured by function-based endpoints alone [68, 69]. Critically reflecting, this limitation also addresses the results of studies using CGA as a cornerstone to initiate GR. Within these studies CGA is mentioned, but no further information on detailed assessments used as “CGA-toolbox” was provided. Only few studies used patient-related outcome measures (PROMs) such as the EQ-5D [25–28, 33, 38, 49, 51], although literature shows that using these measures is a promising approach for value-based healthcare [70–73]. Taken together, our findings point to an important implication for GR practice: the need for consensus on a core toolbox of CGA and goal-setting instruments that can support a high-quality, patient-centred GR process [71]. Anchored in the biopsychosocial model and aligned with contemporary patient-centred care frameworks, such a toolbox would support comprehensive assessment at admission, facilitate individualised and shared goal-setting, enable meaningful monitoring and evaluation of rehabilitation outcomes, and improve continuity across transitions of care. In addition, the findings underscore the necessity of incorporating a core set of evidence-based PROMs that capture older people’s priorities, experiences and perceived benefits of GR, thereby complementing function-based measures with outcomes that reflect what matters most to patients and their ability to participate in life [74]. The work of Festen, Lubbe and van der Klei et al. [66–68] could merely serve as a starting point for such innovation.

Another finding was the marked heterogeneity in terminology used across the studies, regarding the description of patients, structure as well as the GR-process, which is in line with a previous publication [75]. Across studies, terms such as inpatient, post-acute GR, intermediate geriatric rehabilitative care, home-based, and ambulatory GR are often used interchangeably, despite meaningful differences in service organisation, duration, therapeutic intensity, and patient characteristics [76]. This semantic variability complicates the interpretation and comparison of results and limits the transferability. Recent international consensus efforts underscore the need for harmonised terminology, particularly as GR increasingly spans diverse settings and healthcare systems [75]. Overall, the persistent inconsistency in terminology suggests not merely linguistic variation, but a more fundamental lack of shared understanding of key cornerstones of GR, with important implications for research synthesis, outcome measurement, and clinical implementation.

This systematic review demonstrates that to date, no RCTs or controlled prospective primary cohort studies have evaluated GR as a complete care process. However, the excluded qualitative, mixed-methods, and implementation-focused studies (see Supplementary File 2) provide complementary insights into the rehabilitation process, particularly regarding shared decision-making, collaborative goal-setting, patient participation, caregiver involvement, interprofessional communication, structured multidisciplinary team meetings, accessibility, and continuity of care. These elements are not necessarily tested as isolated interventions in controlled trials, but they appear to represent important mechanisms through which rehabilitation components are delivered, adapted, and sustained. Therefore, our findings should be interpreted as identifying outcome-linked core components, while the broader process literature helps contextualise how these components may be implemented within patient-centred GR.

Studying the full GR process within a randomised-controlled design is inherently complex, as it requires integration across care settings and the use of shared, pre-defined and consensual terminology and process quality indicators, including assessment, patient-centred goal-setting, intervention planning and outcome evaluation. Importantly, this complexity also extends to outcome measurement, as meaningful evaluation of the GR process depends on the inclusion of patient-centred outcomes that capture patients’ priorities and experiences across phases of care, and integrated care aspects for GR. Retrospective or qualitative studies, for example, on healthcare professionals’ shared decision-making competencies, active participation in therapy sessions with improved functional outcomes, person-centred, continuous support for frail older adults, improved access and tailored group programmes, barriers such as fear of falling, and interprofessional coordination during training in activities of daily living could help refine process components for future RCTs [77–80]. In this context, the WHO Rehabilitation initiative and the Integrated Care for Older People (ICOPE) pathway at least provide some conceptual guidance, particularly for defining transition criteria between phases of GR and the opportunity to align outcome measurement with a patient-centred, process-based approach [1–3].

Finally, it remains unclear whether comprehensive care and support delivered in less intensive settings, such as the home environment or residential care facilities, may represent a more appropriate and effective approach for selected older, multimorbid and especially frail people [36, 81]. While the available evidence is limited, findings indicate the effectiveness and feasibility of outpatient or home-based models of GR in targeted populations, suggesting that setting selection should be informed by integrated care and pathway-based considerations rather than institutional availability alone. Consistent with the discussion on outcome measurement, cognitive status emerges as a key contextual factor shaping both the choice of GR setting and rehabilitation processes. Severe dementia has been associated with poorer rehabilitation outcomes, whereas higher therapy intensity and improvements in cognitive functioning appear to support rehabilitation effectiveness [35, 38, 50]. Importantly, cognitive impairment alone should not constitute a sole criterion for exclusion from GR [6]. Instead, clear, patient-centred guidance is required to delineate the levels and profiles of dementia for which inpatient GR remains appropriate, and to ensure alignment between GR pathways, transitions across settings, and outcomes in the activity and participation level that matter to older people.

## Strengths

One strength of this study is the comprehensive search strategy and the comparable background of the authors, which led to less need for discussion during the review process and to a common understanding. This systematic review provides a broad overview of contemporary approaches to GR, drawing on a diverse body of studies from multiple countries and GR care settings. By addressing key elements of the GR process, including admission, GR delivery based on assessments and individual goal-setting, therapy composition, discharge- and transition planning, it offers valuable insight into how diverse GR is currently conceptualised and delivered.

## Limitations

A limitation of this review is that the eligibility criteria prioritised comparative intervention studies and may therefore have underrepresented qualitative, implementation-focused, and process-descriptive literature relevant to the operationalisation of GR care processes.

Independent from the methodology possibly biasing data, the heterogeneity of study designs, interventions and outcome measures limits comparability and weakens conclusions regarding GR process design and related quality markers. While the findings consistently highlight the importance of individualised GR aligned with patient-centred care principles based on the biopsychosocial model, they also reveal substantial gaps in standardisation, pathway definitions, monitoring and outcome measurement. These limitations reinforce the need to conceptualise GR as an integrated, pathway-based intervention supported by agreed assessment frameworks and patient-centred, participation-oriented outcome measures, rather than as a collection of isolated interventions. The selection of study designs should also be considered, as RCTs, although providing the highest level of evidence, may be difficult to conduct in process-related GR research. In addition, review-related limitations should be acknowledged. The broad search strategy resulted in many records being excluded during title and abstract screening, suggesting that a more targeted approach may have improved efficiency. Studies may also have been missed due to inconsistent terminology. Finally, restricting inclusion to RCTs and prospective cohort studies may have excluded informative quasi-experimental or implementation-focused research relevant to real-world GR delivery.

## Conclusion

Across diverse settings and study designs, studies on GR included in this review consistently report care process components. However, these components are not embedded within reproducible process structures, as studies show heterogeneous depth of implementation, variable team compositions and used assessments. This variability limited standardised grouping and comparison of collected information and highlights fragmentation in the description and implementation of GR care processes.

The absence of clearly defined core components and standardised process indicators restricts the comparability, implementation, and evaluation of GR. Future research should prioritise study designs that span the full GR pathway across settings, incorporate standardised process indicators, a toolbox of CGA and goal-setting instruments, and evaluate outcomes that reflect the biopsychosocial principles of the ICF.

## Supplementary Information

Below is the link to the electronic supplementary material.

Supplementary file1 (DOCX 25 KB)

Supplementary file2 (DOCX 216 KB)

Supplementary file3 (DOCX 222 KB)

Supplementary file4 (PDF 99 KB)

Supplementary file5 (PDF 83 KB)

## Data Availability

The author confirms that all data generated or analysed during this study are included in this published article and the supplementary material.
