# Supplementary material for: Current evidence on the core components of the geriatric rehabilitation process: a systematic review
Source: Aging Clin Exp Res. 2026 Jul 15;38(1):212. doi: 10.1007/s40520-026-03450-z (PMC13672504; doi:10.1007/s40520-026-03450-z)
Supplement: Supplementary file 1 [file 40520_2026_3450_MOESM4_ESM.pdf]

Study	Title	Exclusion reason
Bencivenga 2022	Role of frailty on cardiac rehabilitation in hospitalized older patients.	Wrong study design / publication type
Loveland 2022	Geriatric home-based rehabilitation in Australia: Preliminary data from an inpatient bed-substitution model.	Wrong study design / publication type
Arjunan 2018	Feasibility and validity of frailty measurement in geriatric rehabilitation.	Wrong study design / publication type
Soh 2023	Clinical frailty scale score during geriatric rehabilitation predicts short-term mortality: RESORT cohort study.	Wrong study design / publication type
Soh 2021	Trajectories of functional performance recovery after inpatient geriatric rehabilitation: an observational study.	Wrong study design / publication type
Morghen 2017	The association between patient participation and functional gain following inpatient rehabilitation.	Wrong study design / publication type
Tedesco 2018	Impact of rehabilitation on mortality and readmissions after surgery for hip fracture.	Wrong study design / publication type
VerstraetenLMG 2024	The impact of mobility limitations on geriatric rehabilitation outcomes: Positive effects of resistance exercise training (RESORT).	Wrong study design / publication type
Rose 2019	Shared decision-making within goal-setting in rehabilitation: a mixed-methods study.	Wrong study design / publication type
Pfeufer 2020	Multidisciplinary inpatient rehabilitation improves the long-term functional status of geriatric hip-fracture patients.	Wrong study design / publication type
Soh 2022	Predictors for the Transitions of Poor Clinical Outcomes Among Geriatric Rehabilitation Inpatients.	Wrong study design / publication type
Low 2021	Impact of place of residence frailty and other factors on rehabilitation outcomes post hip fracture.	Wrong study design / publication type
Johansson 2023	Frail community-dwelling older persons' everyday lives and their experiences of rehabilitation - a qualitative study.	Wrong study design / publication type
Goos-Peek 2017	The added value of clinical geriatric assessment prior to geriatric rehabilitation.	Wrong study design / publication type
Kool 2017	Predictors for living at home after geriatric inpatient rehabilitation: A prospective cohort study.	Wrong study design / publication type
Honda 2024	Impact of frailty and prefrailty on the mid-term outcomes and rehabilitation course after cardiac surgery.	Wrong intervention
Beck 2017	[Focus on nutrition improves the effect of rehabilitation in geriatric patients].	Wrong language
Arjunan 2019	Gait Speed and Frailty Status in Relation to Adverse Outcomes in Geriatric Rehabilitation.	Wrong study design / publication type
TolleyAPL 2022	Characteristics of geriatric rehabilitation inpatients based on their frailty severity and change in frailty severity during admission: RESORT.	Wrong study design / publication type
Ohta 2021	Predicting factors of elderly patients' discharge to home after rehabilitation in rural Japan: a retrospective cohort study.	Wrong study design / publication type
vanTol 2024	Post-COVID-19 patients in geriatric rehabilitation substantially recover in daily functioning and quality of life.	Wrong study design / publication type
Williams 2024	An Early Supported Discharge (ESD) Model of Care for Older Adults Admitted to Hospital: A Descriptive Cohort Study.	Wrong study design / publication type

VluggenTPMM 2020	Factors associated with successful home discharge after inpatient rehabilitation in frail older stroke patients.	Wrong study design / publication type
Ullrich 2019	Life-space mobility in older persons with cognitive impairment after discharge from geriatric rehabilitation.	Wrong study design / publication type
Jorgensen 2020	A cohort study of the effects of multidisciplinary in-patient primary care in older adults.	Wrong study design / publication type
Eichler 2020	Geriatric or cardiac rehabilitation? Predictors of treatment pathways in advanced age patients after transcatheter aortic valve implantation.	Wrong study design / publication type
Marston 2022	Is there a relationship between intensity of occupational therapy and functional outcomes in hospitalised older patients? A prospective cohort study.	Wrong study design / publication type
Rinaldo 2022	Functional capacity assessment and Minimal Clinically Important Difference in post-acute cardiac patients: the role of Short Physical Performance Battery.	Wrong study design / publication type
Ogawa 2022	Poor Oral Health Is a Factor that Attenuates the Effect of Rehabilitation in Older Male Patients with Fractures.	Wrong study design / publication type
Hauer 2017	Effects of Standardized Home Training in Patients with Cognitive Impairment following Geriatric Rehabilitation: A Randomized Controlled Pilot Study.	Wrong intervention
Schuijt 2021	Does the frailty index predict discharge disposition and length of stay at the hospital and rehabilitation facilities?	Wrong study design / publication type
Stuck 2022	Ability of 3 Frailty Measures to Predict Short-Term Outcomes in Older Patients Admitted for Post-Acute Inpatient Rehabilitation.	Wrong study design / publication type
Yamashita 2022	Overlapping states of AWGS muscle dysfunction and inverse feasibility of ADL recovery by rehabilitation in older inpatients.	Wrong study design / publication type
Singh 2012	Predictors of adverse outcomes on an acute geriatric rehabilitation ward.	Wrong study design / publication type
VluggenTPMM 2020	Feasibility of an integrated multidisciplinary geriatric rehabilitation programme for older stroke patients: a process evaluation.	Wrong study design / publication type
Hershkovitz 2019	The association between hand grip strength and rehabilitation outcome in post-acute hip fractured patients.	Wrong study design / publication type
Ni 2017	The rehabilitation enhancing aging through connected health (REACH) study: study protocol for a quasi-experimental clinical trial.	Wrong study design / publication type
Bachmann 2018	Association of potentially inappropriate medications with outcomes of inpatient geriatric rehabilitation : AÂ prospective cohort study.	Wrong study design / publication type
Ullrich 2022	Effects of a Home-Based Physical Training and Activity Promotion Program in Community-Dwelling Older Persons with Cognitive Impairment after Discharge from Rehabilitation: A Randomized Controlled Trial.	Wrong intervention
Dutzi 2017	Cognitive Change in Rehabilitation Patients with Dementia: Prevalence and Association with Rehabilitation Success.	Wrong study design / publication type
Takatori 2023	Effects of social activity participation and trust in the community on the transition of frailty classification in late-stage older adults: a 4-year prospective cohort study.	Wrong study design / publication type
Soh 2022	Comparison of the modified Frailty-Index based on laboratory tests and the Clinical Frailty Scale in predicting mortality among geriatric rehabilitation inpatients: RESORT.	Wrong study design / publication type
Coleman 2012	Outcomes among older people in a post-acute inpatient rehabilitation unit.	Wrong study design / publication type
Moen 2018	Physical function of elderly patients with multimorbidity upon acute hospital admission versus 3â€‰weeks post-discharge.	Wrong study design / publication type
Panzeri 2021	Gender differences and long-term outcome of over 75 elderlies in cardiac rehabilitation: highlighting the role of psychological and physical factors through a secondary analysis of a cohort study.	Wrong intervention

Shimizu 2024	Relationship between independence in activities of daily living at discharge and physical activity at admission of older postoperative hip fracture rehabilitation inpatients: A retrospective case-control study.	Wrong study design / publication type
Lelli 2019	25(OH) vitamin D and functional outcomes in older adults admitted to rehabilitation units: the safari study.	Wrong study design / publication type
Mitsutake 2020	Associations of Hospital Discharge Services With Potentially Avoidable Readmissions Within 30 Days Among Older Adults After Rehabilitation in Acute Care Hospitals in Tokyo; Japan.;"	Wrong study design / publication type
Wilhelmson 2011	Design of a randomized controlled study of a multi-professional and multidimensional intervention targeting frail elderly people.	Wrong study design / publication type
Kiel 2019	[Course of Treatment and Sustainability of Ambulatory Geriatric Rehabilitation - An Analysis of 128 Geriatric Patients].	Wrong language
Fairhall 2011	ICF participation restriction is common in frail - community-dwelling older people: an observational cross-sectional study.	Wrong study design / publication type
Chrusciel 2024	Predictive value of a self-administered frailty screening questionnaire for the effectiveness of functional rehabilitation evaluated with the locomotor functional independence measure in a geriatric rehabilitation unit: a multicentre cohort	Wrong study design / publication type
Calle 2018	Frailty Related Factors as Predictors of Functional Recovery in Geriatric Rehabilitation: The Sarcopenia And Function in Aging Rehabilitation (SAFARI) Multi-Centric study.	Wrong study design / publication type
Ogawa 2021	Elderly Patients after Stroke Increase Skeletal Muscle Mass by Exercise Therapy in Rehabilitation Wards.	Wrong study design / publication type
Guido 2015	A comorbidity prognostic effect on post-hospitalization outcome in a geriatric rehabilitation setting: the pivotal role of functionality - assessed by mediation model - and association with the Brass index.	Wrong study design / publication type
Thalmann 2020	Do older adults benefit from post-acute care following hospitalisation? A prospective cohort study at three Swiss nursing homes.	Wrong study design / publication type
Schoon 2014	Gait speed as a test for monitoring frailty in community-dwelling older people has the highest diagnostic value compared to step length and chair rise time.	Wrong study design / publication type
Roberts 2012	Is grip strength associated with length of stay in hospitalised older patients admitted for rehabilitation? Findings from the Southampton grip strength study.	Wrong study design / publication type
Jamour 2014	[Effectiveness of geriatric rehabilitation in the oldest old: evaluation of South German observational data].	Wrong language
Parker 2015	Randomized controlled trial of the effect of additional functional exercise during slow-stream rehabilitation in a regional center.	Wrong intervention
VanceaNemirschi 2024	Exploring the Connections Between Grip Strength - Nutritional Status - Frailty - Depression - and Cognition as Initial Assessment Tools in Geriatric Rehabilitation-A Pilot Study.	Wrong study design / publication type
VigorÃ¨ 2022	Frailty and rehabilitation outcome in older patients with cardiorespiratory disease: preliminary multidimensional data.	Wrong study design / publication type
Tuna 2019	Rapid Geriatric Assessment - Physical Activity - and Sleep Quality in Adults Aged more than 65 Years: A Preliminary Study.	Wrong study design / publication type
Ogawa 2022	Independent factors that attenuate the effectiveness of fracture rehabilitation in improving activities of daily living in female patients aged 80Â years and above.	Wrong study design / publication type
Yoo 2013	Intensified rehabilitation therapy and transitions to skilled nursing facilities in community-living seniors with acute medical illnesses.	Wrong intervention
Kiel 2020	Evaluation of an ambulatory geriatric rehabilitation program - results of a matched cohort study based on claims data.	Wrong study design / publication type
Aickin 2022	Prevalence of frailty and frailty outcomes within the inpatient rehabilitation setting: use of routinely collected electronic health information.	Wrong study design / publication type
Vega-RamÃ-rez 2017	Analysis of home-based rehabilitation in patients with motor impairment in primary care: a prospective observational study.	Wrong study design / publication type

Ushijima 2021	Effects of cardiac rehabilitation on physical function and exercise capacity in elderly cardiovascular patients with frailty.	Wrong intervention
Pereira 2010	How can we improve targeting of frail elderly patients to a geriatric day-hospital rehabilitation program?	Wrong study design / publication type
Budui 2019	Effects of an Intensive Inpatient Rehabilitation Program in Elderly Patients with Obesity.	Wrong study design / publication type
GonzÃ¡lez-Bernal 2021	Influence of the Use of Wii Games on Physical Frailty Components in Institutionalized Older Adults.	Wrong intervention
Baldasseroni 2023	Predictors of physical frailty improvement in older patients enrolled in a multidisciplinary cardiac rehabilitation program.	Wrong study design / publication type
Drenth 2023	Nursing home geriatric rehabilitation care and interprofessional collaboration; a practice-based study.	Wrong study design / publication type
Yamashita 2023	Protocols and Features of Goal-Setting-Based Intervention for Frail Older Adults in Community Exercise Facilities.	Wrong study design / publication type
Comans 2011	Factors associated with quality of life and caregiver strain amongst frail older adults referred to a community rehabilitation service: implications for service delivery.	Wrong study design / publication type
Pacifico 2022	The Association between Sarcopenia as a Comorbid Disease and Incidence of Institutionalisation and Mortality in Geriatric Rehabilitation Inpatients: REStORing health of acutely unwell adulTs (RESORT).	Wrong study design / publication type
Shimizu 2024	Effects of Sarcopenia on Changes in the Prevalence of Patients with Depressive Mood during Inpatient Geriatric Rehabilitation.	Wrong study design / publication type
Sasaki 2024	Implementation status of postoperative rehabilitation for older patients with hip fracture in Kyoto City - Japan: A population-based study using medical and long-term care insurance claims data.	Wrong study design / publication type
Hettiarachchi 2024	Body Weight and Composition Changes in Geriatric Rehabilitation Are Dependent on Sarcopenia and Malnutrition: RESORT.	Wrong study design / publication type
Daniel 2012	Wii-hab for pre-frail older adults.;"	Wrong intervention
Shimizu 2023	Early physical rehabilitation effectiveness in frail older patients hospitalized for community-acquired pneumonia: analysis of a nationwide database in Japan.	Wrong study design / publication type
Pongpipatpaiboon 2018	Preliminary Study on Prevalence and Associated Factors with Sarcopenia in a Geriatric Hospitalized Rehabilitation Setting.	Wrong study design / publication type
Steinmetz 2024	The prevalence and impact of sarcopenia in older cardiac patients undergoing inpatient cardiac rehabilitation - results from a prospective - observational cohort pre-study.	Wrong study design / publication type
Niccoli 2017	Whey Protein Supplementation Improves Rehabilitation Outcomes in Hospitalized Geriatric Patients: A Double Blinded - Randomized Controlled Trial.	Wrong population
deGroot 2023	Association of Vulnerability Screening on Hospital Admission with Discharge to Rehabilitation-Oriented Care after Acute Hospital Stay.	Wrong study design / publication type
Lindemann 2012	Physical activity of moderately impaired elderly stroke patients during rehabilitation.	Wrong study design / publication type
TseMMY 2020	Frailty is associated with pain and cognitive function in older people in post-acute care settings.	Wrong study design / publication type
Ramezani 2019	A Combination of Indoor Localization and Wearable Sensor-Based Physical Activity Recognition to Assess Older Patients Undergoing Subacute Rehabilitation: Baseline Study Results.	Wrong intervention
RincÃ© 2021	Impact of an individual personalised rehabilitation program on mobility performance in older-old people.	Wrong study design / publication type
Harada 2017	Effectiveness of Cardiac Rehabilitation for Prevention and Treatment of Sarcopenia in Patients with Cardiovascular Disease - A Retrospective Cross-Sectional Analysis.	Wrong study design / publication type

Adachi 2022	Association among mild cognitive impairment - social frailty - and clinical events in elderly patients with cardiovascular disease.	Wrong study design / publication type
Ogawa 2022	Older patients with less skeletal muscle mass gain more skeletal muscle in rehabilitation wards after fractures.	Wrong study design / publication type
Peel 2013	Impact of Post-Acute Transition Care for Frail Older People: A Prospective Study.	Wrong study design / publication type
Corna 2021	Addition of aerobic training to conventional rehabilitation after hip fracture: a randomized - controlled - pilot feasibility study.	Wrong intervention
vanDamvanIsselt 2018	Geriatric rehabilitation in older patients with cardiovascular disease: a feasibility study.	Wrong study design / publication type
Toft 2024	Feasibility of early digital health rehabilitation after cardiac surgery in the elderly: a qualitative study.	Wrong study design / publication type
Hauer 2020	Specific Motor and Cognitive Performances Predict Falls during Ward-Based Geriatric Rehabilitation in Patients with Dementia.	Wrong study design / publication type
Ogawa 2022	Factors associated with actual skeletal muscle mass increase during hip fracture rehabilitation of persons aged 80 and older.	Wrong study design / publication type
Shiozaki 2023	Effects of Physiatrist and Physiotherapist-supervised Therapeutic Exercise on Physical Function in Frail Older Patients with Multimorbidity.	Wrong study design / publication type
Ramsey 2021	Geriatric Rehabilitation Inpatients Roam at Home! A Matched Cohort Study of Objectively Measured Physical Activity and Sedentary Behavior in Home-Based and Hospital-Based Settings.	Wrong study design / publication type
Freitas 2021	Outpatient and Home-Based Treatment: Effective Settings for Hip Fracture Rehabilitation in Elderly Patients.	Wrong study design / publication type
Yoshimura 2022	Pharmacotherapy and the Role of Pharmacists in Rehabilitation Medicine.	Wrong study design / publication type
JÃ¸rgensen 2022	Prevalence of Computer Use among Geriatric In- and Outpatients.	Wrong study design / publication type
Guerzoni 2020	A two-year multicenter point prevalence study of older patients with hip fractures admitted to rehabilitation units in Italy.	Wrong study design / publication type
MagalhÃ£es 2023	Implementation of Orthogeriatrics in Portugal.	Wrong study design / publication type
Zuilekom 2019	[Geriatric rehabilitation of patients selected at the emergency department: patient factors related to rehabilitation outcome].	Wrong language
Cooper 2020	Prehabilitation vs Postoperative Rehabilitation for Frail Patients.	Wrong study design / publication type
LÃ³pez-GarcÃ-a 2024	Telerehabilitation: Vestibular Physiotherapy vs. Multicomponent Exercise for Functional Improvement in Older Adults: Randomized Clinical Trial.	Wrong intervention
Romero-Ortuno 2014	The Impact of Frailty on Post-Acute Rehabilitation Outcomes in Older Adults.	Wrong study design / publication type
Hewage 2023	The Hospital to Home study (H2H): smartwatch technology-enabled rehabilitation following hip fracture in older adults - a feasibility non-randomised trial.	Wrong study design / publication type
Yoshimura 2022	Chair-Stand Exercise Improves Sarcopenia in Rehabilitation Patients after Stroke.	Wrong study design / publication type
Maiden 2024	Reimagining Day Rehabilitation For Frailty and Neurodegenerative Conditions through the integrated Rehabilitation and EnAblement Program (iREAP).	Wrong study design / publication type
RaijmannRCMA 2024	Impact of geriatric co-management on outcomes in hospitalised cardiology patients aged 85Â and over.	Wrong study design / publication type

VerstraetenLMG 2023	Sarcopenia - malnutrition and cognition affect physiotherapy frequency during geriatric rehabilitation: RESORT cohort.	Wrong study design / publication type
Brocki 2023	Exercise-based real-time telerehabilitation for older patients recently discharged after transcatheter aortic valve implantation: An extended feasibility study.	Wrong study design / publication type
EverinkIHJ 2023	Nutritional Care Practices in Geriatric Rehabilitation Facilities across Europe: A Cross-Sectional Study.	Wrong study design / publication type
Suzuki 2024	Evaluation of a novel sub-acute Hospital in the Home model for providing inpatient geriatric and rehabilitation services.	Wrong study design / publication type
Chang 2025	The Usability and Effect of a Novel Intelligent Rehabilitation Exergame System on Quality of Life in Frail Older Adults: Prospective Cohort Study.	Wrong study design / publication type
Keilhauer 2024	[Rehabilitative subacute inpatient care-Optimizing posthospital care for geriatric patients with rehabilitation needs: results of the REKUP study].	Wrong language
FariaADCA 2023	Effect of the Active Aging-in-Place-Rehabilitation Nursing Program: A Randomized Controlled Trial.	Wrong intervention
Bayon-Calatayud 2018	Short-Term Outcomes of Interdisciplinary Hip Fracture Rehabilitation in Frail Elderly Inpatients.	Wrong study design / publication type
Rundell 2024	Associations of Co-Occurring Chronic Conditions With Use of Rehabilitation Services in Older Adults With Back Pain: A Population-Based Cohort Study.	Wrong study design / publication type
Krakers 2024	Rehabilitation of Frail Older Adults after Hip Fracture Surgery: Predictors for the Length of Geriatric Rehabilitation Stay at a Skilled Nursing Home.	Wrong study design / publication type
Krumov 2019	The benefits to functional ambulation and physical activity of group-based rehabilitation in frail elderly Bulgarians undergoing total knee arthroplasty. Preliminary results.	Wrong study design / publication type
Pedroli 2022	Brain M-App's Structure and Usability: A New Application for Cognitive Rehabilitation at Home.	Wrong study design / publication type
Lubovsky 2024	Perioperative and Postoperative Outcomes of Proximal Hip Fracture: A Comparison of Orthopedic and Geriatric Care Models.	Wrong study design / publication type
Steinmetz 2024	Evaluation of frailty in geriatric patients undergoing cardiac rehabilitation after cardiac procedure: results of a prospective - cross-sectional study.	Wrong study design / publication type
VlÄƒduÈ›u 2024	Assessment and Rehabilitation in Sarcopenic Patients.	Wrong study design / publication type
Hashimoto 2022	Benefits of a Balance Exercise Assist Robot in the Cardiac Rehabilitation of Older Adults with Cardiovascular Disease: A Preliminary Study.	Wrong study design / publication type
Honzawa 2020	Relationship Between Kihon Checklist Score and Anxiety Levels in Elderly Patients Undergoing Early Phase II Cardiac Rehabilitation.	Wrong study design / publication type
Lee 2025	Outcomes after inpatient rehabilitation for older adults with hip fractures: A cross-sectional study.	Wrong study design / publication type
Noeske 2025	Walking self-confidence and lower levels of anxiety are associated with meeting recommended levels of physical activity after hip fracture: a cross-sectional study.	Wrong intervention
Urquiza 2025	Predictors of Hospital Readmission - Institutionalization and Mortality in Geriatric Rehabilitation Following Hospitalization According to Admission Reason.	Wrong study design / publication type
NÃºÃ±ez-CortÃ©s 2024	Comorbidity burden and nutritional status are associated with short-term improvement in functional independence and pain intensity after hip fracture surgery in older adults with in-hospital rehabilitation.	Wrong study design / publication type
Deutsch 2024	Trends in the Characteristics and Outcomes of Older Medicare Patients With Traumatic Brain Injury Treated in Inpatient Rehabilitation Facilities: 2013 to 2018.	Wrong study design / publication type
Schroeder 2024	Home Versus Hospital Rehabilitation of Older Adults Following Hip Fracture Yields Similar Patient-Reported Outcome Measures.	Wrong study design / publication type

Pattath 2023	A Comparison of Acute Ischemic Stroke Patients Discharged to Inpatient Rehabilitation vs a Skilled Nursing Facility: The Paul Coverdell National Acute Stroke Program.	Wrong study design / publication type
Hayes 2023	Medicare Advantage Patterns of Poststroke Discharge to an Inpatient Rehabilitation or Skilled Nursing Facility: A Consideration of Demographic - Functional and Payer Factors.	Wrong study design / publication type
Snowdon 2023	Client and service factors associated with changes in health-related quality of life following community rehabilitation.	Wrong study design / publication type
Nishiomasu 2023	Indicators of Improvement in Performing Activities of Daily Living Among Older Patients Undergoing Rehabilitation Following Hip Fractures.	Wrong study design / publication type
Andersen 2022	Impact of Frailty on Acute Rehabilitation Outcomes: An Observational Study in a Regional Australian Context.	Wrong study design / publication type
Hershkovitz 2022	Are extracapsular and intracapsular hip-fracture patients two distinct rehabilitation subpopulations?	Wrong study design / publication type
Morri 2022	Post-hospital care pathway for individuals with hip fracture: what is the optimal setting and rehabilitation intensity? An observational study.	Wrong study design / publication type
Schuetze 2022	Geriatric patients with dementia show increased mortality and lack of functional recovery after hip fracture treated with hemiprosthesis.	Wrong study design / publication type
Lindblom 2022	Referral-based transition to subsequent rehabilitation at home after stroke: one-year outcomes and use of healthcare services.	Wrong study design / publication type
Ohtsubo 2022	Association of sarcopenia and physical activity with functional outcome in older Asian patients hospitalized for rehabilitation.	Wrong study design / publication type
Peiris 2022	Functional status of community-dwelling older adults after inpatient rehabilitation.	Wrong study design / publication type
Nishioka 2021	Prevalence and Associated Factors of Coexistence of Malnutrition and Sarcopenia in Geriatric Rehabilitation.	Wrong study design / publication type
Simpson 2021	New Problems and Iatrogenic Events Among Older Adults in the First 30 Days of Post-Acute Rehabilitation.	Wrong study design / publication type
Wan 2021	Risk Factors of Readmissions in Geriatric Rehabilitation Patients: RESORT.	Wrong study design / publication type
Morris 2021	Demographic Characteristics and Functional Levels of Patients With Fragility Fractures Who Accept Tele-rehabilitation as an Alternative to Face-to-face Home Rehabilitation.	Wrong study design / publication type
Verstraeten 2021	Association between malnutrition and stages of sarcopenia in geriatric rehabilitation inpatients: RESORT.	Wrong study design / publication type
Warhurst 2020	Patient characteristics - treatment outcomes and rehabilitation practices for patients admitted with hip fractures using multiple data set analysis.	Wrong study design / publication type
Laurent 2020	Functional decline in geriatric rehabilitation ward; is it ascribable to hospital acquired infection? A prospective cohort study.	Wrong study design / publication type
Daly 2020	Predictors of Exceeding Target Inpatient Rehabilitation Length of Stay After Hip Fracture.	Wrong study design / publication type
Kabboord 2020	The modified functional comorbidity index performed better than the Charlson index and original functional comorbidity index in predicting functional outcome in geriatric rehabilitation: a prospective observational study.	Wrong study design / publication type
McKechnie 2020	Examination of the dependency and complexity of patients admitted to in-patient rehabilitation in Australia.	Wrong study design / publication type
Andrews 2018	Improvement During Inpatient Rehabilitation Among Older Adults With Guillain-BarrÃ© Syndrome - Multiple Sclerosis - Parkinson Disease and Stroke.	Wrong study design / publication type
Cecchi 2018	Predictors of recovering ambulation after hip fracture inpatient rehabilitation.	Wrong study design / publication type

Petrelli 2018	Effect of Socioeconomic Status on Surgery Waiting Times and Mortality After Hip Fractures in Italy.	Wrong study design / publication type
Hershkovitz 2018	The Association between Anticholinergic Drug Use and Rehabilitation Outcome in Post-Acute Hip Fractured Patients: A Retrospective Cohort Study.	Wrong study design / publication type
Hershkovitz 2017	Place orientation and visual construction subdomains of the Mini Mental State Examination test as predictors of rehabilitation outcome of post-acute hip-fractured patients.	Wrong study design / publication type
SUGARHOOD 2017	Participation in advanced age: enacting values - an adaptive process.	Wrong study design / publication type
Huijbenâ€Schoenmakers 2017	Cognition in relation to independency in older - comorbid stroke patients in a stroke unit.	Wrong study design / publication type
Kumar 2017	Comorbidity Indices Versus Function as Potential Predictors of 30-Day Readmission in Older Patients Following Postacute Rehabilitation.	Wrong study design / publication type
Bernard 2016	Higher Charlson Comorbidity Index scores do not influence Functional Independence Measure score gains in older rehabilitation patients.	Wrong study design / publication type
Mitchell 2016	Hip fracture and the influence of dementia on health outcomes and access to hospital-based rehabilitation for older individuals.	Wrong study design / publication type
Liu 2016	Association Between Hospital Admission Risk Profile Score and Skilled Nursing or Acute Rehabilitation Facility Discharges in Hospitalized Older Adults.	Wrong study design / publication type
Galloway 2016	Hospital Readmission Following Discharge From Inpatient Rehabilitation for Older Adults With Debility.	Wrong study design / publication type
Nolan 2016	Frailty and its association with rehabilitation outcomes in a post-acute older setting.	Wrong study design / publication type
Fisher 2016	Predictors of 30-Day Readmission Following Inpatient Rehabilitation for Patients at High Risk for Hospital Readmission.	Wrong study design / publication type
Morandi 2015	The Association Between the Probability of Sarcopenia and Functional Outcomes in Older Patients Undergoing In-Hospital Rehabilitation.	Wrong study design / publication type
Kushner 2015	Evaluating Siebens Domain Management Model for Inpatient Rehabilitation to Increase Functional Independence and Discharge Rate to Home in Geriatric Patients.	Wrong study design / publication type
Ireland 2015	Risk factor profiles for early and delayed mortality after hip fracture: Analyses of linked Australian Department of Veterans' Affairs databases.	Wrong study design / publication type
Chow 2014	Factors and Trade-Offs With Rehabilitation Effectiveness and Efficiency in Newly Disabled Older Persons.	Wrong study design / publication type
Mallinson 2014	Comparison of Discharge Functional Status After Rehabilitation in Skilled Nursing - Home Health and Medical Rehabilitation Settings for Patients After Hip Fracture Repair.	Wrong study design / publication type
Roberts 2014	Risk factors for discharge to an acute care hospital from inpatient rehabilitation among stroke patients.	Wrong study design / publication type
Vermeulen 2013	Time Use of Stroke Patients with Stroke Admitted for Rehabilitation in Skilled Nursing Facilities.	Wrong study design / publication type
Morandi 2013	Predictors of Rehospitalization Among Elderly Patients Admitted to a Rehabilitation Hospital: The Role of Polypharmacy - Functional Status - and Length of Stay.	Wrong study design / publication type
Mathew 2013	Effect of Comorbidity on Functional Recovery After Hip Fracture in the Elderly.	Wrong study design / publication type
Portegijs 2012	Failure to Regain Function at 3 months After Acute Hospital Admission Predicts Institutionalization Within 12 Months in Older Patients.	Wrong study design / publication type
Martin 2012	A longitudinal study of poor sleep after inpatient post-acute rehabilitation: the role of depression and pre-illness sleep quality.	Wrong study design / publication type

CaryJr 2012	Inpatient Rehabilitation Outcomes in Medicare Hip Fracture Patients.;	Wrong study design / publication type
Torpilliesi 2012	Outcomes of Nonagenarian Patients After Rehabilitation Following Hip Fracture Surgery.	Wrong study design / publication type
Martin 2011	Poor Self-Reported Sleep Quality Predicts Mortality within One Year of Inpatient Post-Acute Rehabilitation among Older Adults.	Wrong study design / publication type
Poynter 2011	Does Cognitive Impairment Affect Rehabilitation Outcome?	Wrong study design / publication type
Dudkiewicz 2011	Gender Differences Among Patients With Proximal Femur Fractures During Rehabilitation.	Wrong study design / publication type
Frengley 2011	Influence of age on rehabilitation outcomes and survival in post-acute inpatient cardiac rehabilitation.	Wrong study design / publication type
Young 2011	Longitudinal Functional Recovery After Postacute Rehabilitation in Older Hip Fracture Patients: The Role of Cognitive Impairment and Implications for Long-Term Care.	Wrong study design / publication type
Denkinger 2010	Relationship between fear of falling and outcomes of an inpatient geriatric rehabilitation population -- fear of the fear of falling.	Wrong study design / publication type
Zheng 2025	Effects of Virtual Reality-Based Activities of Daily Living Rehabilitation Training in Older Adults With Cognitive Frailty and Activities of Daily Living Impairments: a Randomized Controlled Trial	Wrong intervention
unknown authors	Geriatrician-performed Comprehensive Geriatric Care in an Outpatient Community Rehabilitation Unit;"	Wrong study design / publication type
Papaioannou 2023	GERAS VIRTUAL FRAILTY REHABILITATION PROGRAM FOR FRAIL OLDER ADULTS: a PILOT RANDOMIZED CONTROLLED TRIAL	Wrong study design / publication type
Vladutu 2023	OUTPATIENT REHABILITATION IN SARCOPENIC PATIENTS	Wrong study design / publication type
Ioannidis 2022	GERAS FRAILTY REHABILITATION AT HOME RANDOMIZED CONTROLLED TRIAL: VIRTUAL BUNDLED CARE FOR SENIORS WHO ARE FRAIL TO BUILD STRENGTH AND RESILIENCE DURING COVID-19	Wrong study design / publication type
Xandri 2022	How the VIVIFRAIL © Multi-Component Exercise Program can improve the functional capacity of older patients admitted to a functional recovery unit	Wrong study design / publication type
Hirashiki 2022	Robotic Exercise Rehabilitation of Older Adults With Cardiovascular Disease Appears Safe and Effective	Wrong intervention
LiC-M 2010	The effectiveness of a comprehensive geriatric assessment intervention program for frailty in community-dwelling older people: a randomized controlled trial	Wrong intervention
Caplan 2006	Does home treatment affect delirium? A randomised controlled trial of rehabilitation of elderly and care at home or usual treatment (The REACH-OUT trial)	Wrong publication date
Prescott 2021	Home-based Extended Rehabilitation for Older people (HERO): study protocol for an individually randomised controlled multi-centre trial to determine the clinical and cost-effectiveness of a home-based	Wrong study design / publication type
no author	Cognitive Intervention and Rehabilitation Outcomes in Hip Fracture Patients;"	Wrong study design / publication type
Ozaki 2017	Training with a balance exercise assist robot is more effective than conventional training for frail older adults	Wrong intervention
Handoll 2021	Multidisciplinary rehabilitation for older people with hip fractures	Wrong study design / publication type
Campbell 2024	Does an Inpatient Rehabilitation Admission Via the Community Prevent Hospital Readmissions in Older Adults? An Integrated Care Initiative	Wrong study design / publication type
OConnell 2024	Determining Inclusion Criteria For Post-Acute Care Rehabilitation - Home Alone Is Not A Barrier To Success	Wrong study design / publication type

Vigore 2024	Frailty in older patients with chronic obstructive pulmonary disease (COPD) undergoing a multidisciplinary rehabilitation	Wrong study design / publication type
Vigore 2023	Frailty and rehabilitation outcome in older patients with cardiorespiratory disease: preliminary multidimensional data	Wrong study design / publication type
Tay 2023	Functional outcomes of COVID-19 patients who underwent acute inpatient rehabilitation and the exploration of the benefits of adjunct robotic therapy and the effects of frailty	Wrong study design / publication type
Fraenkel 2023	Stepped care versus center-based cardiopulmonary rehabilitation for older frail adults living in rural MA: Design of a feasibility randomized controlled trial	Wrong population
Grote 2023	Outcomes of early rehabilitation in elderly patients	Wrong study design / publication type
Kuo 2023	Measurement of 2 Frailty Indicators to Predict Physical Recovery after Rehabilitation in Older Patients Admitted to Acute Geriatric Ward	Wrong study design / publication type
Magner 2023	Avoiding acute hospital admission through rapid access rehabilitation pathways for frail older patients through cross site integrated working	Wrong study design / publication type
Jain 2023	Social Vulnerability and Delivery of In-hospital Rehabilitation Services to Older Adults With Critical Illness	Wrong study design / publication type
Coen 2022	OLDER PERSONS REHAB AT HOME (OPRAH) INDICATES AN EFFECTIVE ALTERNATIVE PATHWAY TO INPATIENT REHABILITATION	Wrong study design / publication type
Hirashiki 2022	Effects of Cardiac Rehabilitation after Discharge on Frailty and Balance in Elderly Patients with Cardiovascular Disease	Wrong study design / publication type
Haaksma 2022	Recovery of daily functioning and quality of life in post- COVID-19 patients in geriatric rehabilitation	Wrong study design / publication type
Hettiarachchi 2021	Malnutrition is associated with poor trajectories of activities of daily living in geriatric rehabilitation inpatients: RESORT	Wrong study design / publication type
Amata 2021	Role of frailty in functional recovery after hip fracture; the variable impact in restoring autonomy;	Wrong study design / publication type
Cheung 2020	Pilot study on a new intervention programme for geriatric hip fracture patient with sarcopenia	Wrong study design / publication type
Zintchouk 2019	Impact of Geriatrician-Performed Comprehensive Geriatric Care on Medication Use and Cognitive Function in Older Adults Referred to a Non-Hospital-Based Rehabilitation Unit	Wrong outcome
Aizen 2019	Cognitive and functional outcomes in elderly patients with post-stroke delirium	Wrong study design / publication type
DeGroot 2019	Patient characteristics as early indicators of geriatric rehabilitation	Wrong study design / publication type
McDaid 2019	The feasibility of an interdisciplinary led community based functional training group for frail older adults in a post-acute orthogeriatric unit	Wrong study design / publication type
Ho 2019	Pilot study on a new intervention program for geriatric hip fracture patient with sarcopenia	Wrong study design / publication type
Terbraak 2019	Feasibility of home-based cardiac rehabilitation in frail patients	Wrong study design / publication type
Jensen 2019	CGA in rehabilitation units	Wrong study design / publication type
McDaid 2019	Profiling clinical frailty in older adults in an Irish post-acute care ortho-geriatric unit	Wrong study design / publication type
Juul-Larsen 2018	Patterns of multimorbidity in older medical patients (>= 65 years): And how they relate to mobility the first year after an acute admission	Wrong study design / publication type

Markle-Reid 2018	Implementation and evaluation of transitional care intervention linking hospital-based outpatient rehabilitation and community-based care for older adults with stroke and multiple chronic conditions	Wrong study design / publication type
Kavanagh 2017	Early mdt intervention and improved care pathways for the frail older person in a rehabilitation hospital	Wrong study design / publication type
Huded 2015	Frailty status and outcomes following trans-catheter aortic valve replacement	Wrong study design / publication type
Blindenbach 2015	Reduction of hospital admissions after pulmonary rehabilitation of geriatric patients with COPD	Wrong study design / publication type
Nolan 2014	Frailty and its association with rehabilitation outcomes: A prospective cohort study of a post-acute frail older population	Wrong study design / publication type
Power 2013	Development of a 36 bed gerontological rehabilitation unit: A profile of client and hospital outcomes	Wrong study design / publication type
Bertone 2012	New technologies applied to rehabilitation in elderly patients: A pilot study on the step training system	Wrong study design / publication type
Martin 2012	Improving functional status in rehabilitation	Wrong study design / publication type
Littbrand 2011	Effect of a high-intensity functional exercise program on functional balance: Preplanned subgroup analyses of a randomized controlled trial in residential care facilities	Wrong intervention
Coleman 2011	Outcomes among older people in a post-acute inpatient population	Wrong study design / publication type
Singh 2011	Predictors of adverse outcomes on an acute geriatric rehabilitation ward	Wrong study design / publication type
GlothIII 1999	The frail elderly functional assessment questionnaire: Its responsiveness and validity in alternative settings	Wrong publication date
Meyer 2021	Improved balance and gait ability and basic activities of daily living after comprehensive geriatric care in frail older patients with fractures	Wrong study design / publication type
Bordne 2020	Behavioral and emotional quality of life of patients undergoing inpatient geriatric rehabilitation.	Wrong study design / publication type
Mattiazzo 2023	Geriatric rehabilitation care after hip fracture	Wrong study design / publication type
Eijk 2012	Geriatric rehabilitation of lower limb amputees: a multicenter study	Wrong study design / publication type
Doornebosch 2024	Factors influencing interprofessional collaboration in general and during multidisciplinary team meetings in long-term care and geriatric rehabilitation: a qualitative study	Wrong study design / publication type
Scheffers-Barnhoorn 2022	Coping strategies of older adults with a recent hip fracture within inpatient geriatric rehabilitation	Wrong intervention
Zintchouk 2016	Comprehensive Geriatric Care Versus Standard Care for Elderly Referred to a Rehabilitation Unit â€“ a Randomized Controlled Trial	Wrong study design / publication type
