# Supplementary material for: Current evidence on the core components of the geriatric rehabilitation process: a systematic review
Source: Aging Clin Exp Res. 2026 Jul 15;38(1):212. doi: 10.1007/s40520-026-03450-z (PMC13672504; doi:10.1007/s40520-026-03450-z)
Supplement: Supplementary file 2 [file 40520_2026_3450_MOESM5_ESM.pdf]

Table 1: Risk of bias assessment for RCTs

Study ID	Internal Validity										Statistical Conclusion Validity			Overall risk of bias
	Bias related to selection and allocation			Bias related to administration of intervention/exposure			Bias related to assessment, detection and measurement of the outcome			Bias related to participant retention				
Question	1	2	3	4	5	6	7	8	9	10	11	12	13	
Bernocchi 2019	✓	✓	✓	×	×	✓	✓	✓	✓	✓	✓	✓	✓	low
Bruun 2020	✓	✓	✓	×	×	✓	✓	✓	✓	✓	✓	✓	✓	low
Coleman 2021	×	×	✓	×	×	✓	✓	✓	✓	✓	✓	✓	✓	moderate
Comans 2010	✓	✓	✓	×	×	✓	✓	✓	✓	✓	✓	✓	✓	low
Conroy 2010	✓	✓	✓	×	×	✓	✓	✓	✓	✓	✓	✓	✓	low
Courtney 2012	✓	✓	✓	×	×	✓	✓	✓	✓	✓	✓	✓	✓	low
Crotty 2019	✓	✓	✓	×	×	✓	✓	✓	✓	✓	✓	✓	✓	low
Ercal 2021	✓	✓	✓	×	×	✓	✓	✓	✓	✓	✓	✓	✓	low
Fairhall 2012	✓	✓	✓	×	×	✓	✓	✓	✓	✓	✓	✓	✓	low
Goldberg 2019	✓	✓	✓	×	×	✓	×	✓	✓	~	✓	✓	✓	moderate
Han 2021**	✓	✓	✓	×	×	✓	✓	✓	✓	✓	✓	✓	✓	low
Harwood 2023	✓	✓	✓	×	×	✓	×	✓	✓	~	✓	✓	✓	moderate
Hill 2015	✓	✓	✓	×	×	✓	✓	✓	✓	✓	✓	✓	✓	low
Invernizzi 2019	✓	✓	✓	×	×	✓	×	✓	✓	✓	✓	✓	✓	moderate
Lenze 2019	✓	✓	✓	×	×	✓	✓	✓	✓	✓	✓	✓	✓	low
Magaziner 2019	✓	✓	✓	×	×	×	✓	✓	✓	✓	✓	✓	✓	moderate
Maki 2018	×	✓	✓	×	×	✓	×	✓	✓	✓	✓	✓	✓	moderate
Maki 2021	✓	✓	✓	×	×	✓	✓	✓	✓	✓	✓	✓	✓	low
Milte 2016**	✓	✓	✓	×	×	✓	✓	✓	✓	✓	✓	✓	✓	low
Okpara 2023	✓	✓	✓	×	×	✓	✓	✓	✓	✓	✓	✓	✓	low
Sahota 2017	~	~	✓	~	~	✓	~	✓	✓	✓	✓	✓	✓	moderate
Said 2018***	✓	✓	✓	×	×	×	✓	✓	✓	✓	✓	✓	✓	moderate
Said 2021***	✓	✓	✓	×	×	×	✓	✓	✓	✓	×	✓	✓	moderate
Scheffers-Barnhoorn 2019	✓	✓	✓	×	×	✓	✓	✓	✓	✓	✓	✓	✓	low
Shyu 2013	~	~	✓	×	×	✓	~	✓	✓	✓	✓	✓	✓	moderate
Timmer 2020	✓	✓	✓	✓	×	✓	✓	✓	✓	✓	✓	✓	✓	low
Tseng 2019	✓	✓	✓	✓	×	✓	~	✓	✓	✓	✓	✓	✓	moderate
Vluggen 2021	✓	✓	✓	×	×	✓	✓	✓	✓	✓	✓	✓	✓	low
Westgård 2020****	✓	✓	✓	×	×	✓	×	✓	✓	✓	✓	✓	✓	moderate
Wilhelmson 2022****	✓	✓	✓	×	×	✓	×	✓	✓	✓	✓	✓	✓	moderate
Zintchouk 2018	✓	✓	✓	×	×	✓	✓	✓	✓	✓	✓	✓	✓	low

Table 2: Risk of bias assessment for cohort studies

Study ID	Were the two groups similar and recruited from the same population	Were the exposures measured similarly to assign people to both exposed and unexposed groups	Was the exposure measured in a valid and reliable way	Were confounding factors identified	Were strategies to deal with confounding factors stated	Were the groups/participants free of the outcome at the start of the study	Were the outcomes measured in a valid and reliable way	Was the follow up time reported and sufficient to be long enough for outcomes to occur	Was follow up complete, and if not, were the reasons to loss to follow up described and explored	Were strategies to address incomplete follow up utilized	Was appropriate statistical analysis used	Overall risk of bias
Question	1	2	3	4	5	6	7	8	9	10	11	
Everink 2018*	✓	✓	✓	✓	✓	✓	✓	✓	✓	✓	✓	low
EverinkIHJ 2018*	✓	✓	✓	✓	✓	✓	✓	✓	✓	✓	✓	low
Gregersen 2011	✓	✓	✓	~	~	✓	✓	✓	✓	✓	✓	moderate
Holstege 2017	✓	✓	✓	✓	✓	~	✓	~	✓	✓	✓	moderate
Lee 2012	✓	✓	✓	✓	✓	✓	✓	✓	✓	✓	✓	low

Legend:
✓ yes
× no
~ unclear
n.a. not applicable
* same cohort
