# Supplementary material for: Current evidence on the core components of the geriatric rehabilitation process: a systematic review
Source: Aging Clin Exp Res. 2026 Jul 15;38(1):212. doi: 10.1007/s40520-026-03450-z (PMC13672504; doi:10.1007/s40520-026-03450-z)
Supplement: Supplementary file 3 [file 40520_2026_3450_MOESM1_ESM.docx]

Supplementary File 1

**Searchstrings - Systematic Review**

Review title: Current Evidence on the Core Components of the Geriatric Rehabilitation Process: A Systematic Review

Search conducted: 03. February 2025

**PubMed**

((((((((((((("geriatric patien*") OR (aging[MeSH Terms])) OR (ageing))) OR (aged[MeSH Terms])) OR (elder*)) OR (geriatric*))) OR ("older adul*")) OR ("older people")) OR (aging)) AND ((((((multimorbid*) OR (frailty[MeSH Terms])) OR (frail*)) OR ("multiple comorbid*")) OR ("multiple chronic conditio*")) OR ("frailty syndrome"))) AND (((((((((((("geriatric rehabilitation"[Title/Abstract])) OR (telerehabilitation[Title/Abstract])) OR (tele-rehabilitation[Title/Abstract])) OR (telerehabilitation[MeSH Terms])) OR ("mobile rehabilitation"[Title/Abstract])) OR ("home-based rehabilitation"[Title/Abstract])) OR ("home rehabilitation"[Title/Abstract])) OR ("remote rehabilitation"[Title/Abstract])) OR ("inpatient rehabilitation"[Title/Abstract])) OR ("outpatient rehabilitation"[Title/Abstract])) OR (rehabilitation[Title/Abstract]))) AND (((((((((((((((((((((((((((((((((((((((((((((((((((((((((((((((((((((((((((((((((((((((((((((patient participation[MeSH Terms]) OR (participation)) OR ("patient participation")) OR ("rehabilitation process")) OR ("care process")) OR ("process improvement")) OR ("process management")) OR ("process evaluation")) OR (determination, eligibility[MeSH Terms])) OR ("eligibility determinatio*")) OR ("eligibility requiremen*")) OR ("eligibility criteria")) ) ) OR (care, patient centered[MeSH Terms])) OR ("patient centered")) OR ("patient centred")) OR ("patient-centred")) OR ("patient-centered")) OR ("Activities of Daily Living"[MeSH Terms])) OR ("Activities of Daily Living")) OR ("ADL")) OR ("Quality of Life"[MeSH Terms])) OR ("Quality of Life")) OR ("QoL")) OR ("Geriatric Assessment"[MeSH Terms])) OR ("Geriatric Assessment")) OR ("Comprehensive Geriatric Assessment")) OR ("Frailty Assessment")) OR ("CGA")) OR ("Patient Care Team"[MeSH Terms])) OR ("Patient Care Team")) OR (multidisciplin*)) OR (interdisciplin*)) OR (multiprofessional*)) OR (interprofessional*)) OR ("Team Structure")) OR ("healthcare Team")) OR ("care team")) OR ("rehabilitation team")) OR ("rehabilitation care team")) OR (treatment)) OR (intervention)) OR (therapy)) OR ("treatment process")) OR ("patient discharge")) OR ("discharge planning")) OR ("discharge management")) OR ("Continuity of Patient Care"[MeSH Terms])) OR ("Care Transition")) OR ("Patient Transfer")) OR ("patient transition")) OR ("Continuity of Care")) OR (facilitator*)) OR (barrier*)) OR (assignment)) OR (referral)) OR ("Patient Admission"[MeSH Terms])) OR (admission)) OR ("outcome measure*")) OR (measure*)) OR (evaluation)) OR ("test batter*")) OR ("Patient Reported Outcome Measures"[MeSH Terms])) OR ("Patient Reported Outcome Measures")) OR (PROM)) OR (PROMS)) OR ("Patient Reported Experience Measures")) OR (PREM)) OR (PREMS)) OR (goal-setting)) OR ("goal setting")) OR ("Decision Making, Shared"[MeSH Terms])) OR ("shared decision making")) OR (multicomponent)) OR (individualize*)) OR (SDM)) OR (personalize*)) OR ("core team")) OR (Education[MeSH Terms])) OR ("outcome assessment")) OR ("Outcome and Process Assessment, Health Care"[MeSH Terms])) OR (education)) OR (training)) OR ("Health Resources"[MeSH Terms])) ) OR (resource*)) OR ("Patient Readmission"[MeSH Terms])) OR (readmission)) OR ("care need*")) OR (discharge)) OR (Mortality[MeSH Terms])) OR (mortality)) Filters: from 2010 – 2025

**1711 results**

**CINAHL**

**S1**

( multimorbidity, or comorbidity, or multiple chronic conditions ) OR ( frailty or frail elderly or frail ) OR frailty syndrome

**S2**

(MH "Aged") OR "aged"  OR (MH "Aging") OR "aging" OR (MH "Frail Elderly") OR "frail elderly" OR ( aging or ageing or elderly or older adults or seniors or geriatrics ) OR geriatric patien* OR ( geriatric patients or senior or elderly or aged or older adult ) OR older people

**S3**

(MH "Rehabilitation, Geriatric") OR (MH "Telerehabilitation") OR AB geriatric rehabilitation OR AB ( telerehabilitation or tele-rehabilitation or virtual rehabilitation or remote rehabilitation ) OR AB mobile rehabilitation OR AB ( home-based rehabilitation or in home rehabilitation or home rehabilitation ) OR AB inpatient rehabilitation OR AB outpatient rehabilitation OR AB rehabilitation

**S4**

(MH "Social Participation") OR "social participation" OR (MH "Patient Participation") OR "patient participation" OR participation OR „rehabilitation process“ OR „care process“ OR „process improvement“ OR „process management“ OR „process evaluation“ OR (MH "Eligibility Determination") OR "eligibility determination" OR „eligibility requiremen*“ OR „eligibility criteria“ OR ( (MH "Patient Centered Care") OR "patient centered care" ) OR „patient centered“ OR „patient centred“ OR „patient-centered“ OR „patient-centred“ OR ( (MH "Activities of Daily Living") OR "activities of daily living" ) OR adl OR ( (MH "Quality of Life") OR "quality of life" ) OR qol OR ( (MH "Geriatric Assessment") OR "geriatric assessment" ) OR ( „comprehensive geriatric assessment“ or „frailty assessment“ or cga ) OR (MH "Multidisciplinary Care Team") OR „patient care team“ OR multidisciplin* OR interdisciplin* OR multiprofessional* OR interprofessional* OR „team structure“ OR „healthcare team“ OR „care team“ OR „rehabilitation team“ OR „rehabilitation care team“ OR ( treatment or intervention or therapy ) OR „treatment process“ OR „patient discharge“ OR „discharge planning“ OR „discharge management“ OR ( (MH "Continuity of Patient Care") OR "continuity of patient care" ) OR „care transition“ OR „patient transfer“ OR „patient transition“ OR „continuity of care“ OR facilitator* OR barrier* OR assignment OR referral OR ( (MH "Patient Admission") OR "patient admission" ) OR admission OR „outcome measure*“ OR measure* OR evaluation OR „test batter*“ OR ( (MH "Patient-Reported Outcomes") OR "patient reported outcomes" ) OR „patient reported outcome measures“ OR PROMs OR prom OR ( „patient-reported experience measures“ or prem ) OR Prem OR „goal setting“ OR „goal-setting“ OR (MH "Decision Making, Shared") OR „shared decision making“ OR multicomponent OR individualize* OR individualise* OR personalize* OR personalise* OR sdm OR „core team“ OR ( (MH "Education") OR "education" ) OR training OR resource* OR ressource* OR „care need*“ OR ( (MH "Outcome Assessment") OR "outcome assessment" ) OR „health resources“ OR ( (MH "Readmission") OR "readmission" ) OR „patient readmission“ OR (MH "Process Assessment (Health Care)") OR (MH "Mortality") OR "mortality"

S1 AND S2 AND S3 AND S4

Limiters - Publication Date: 20100101-20251231

Expanders - Apply equivalent subjects

Search modes - Find all my search terms

**1688 results**

**Cochrane Central Register of Controlled Trials and Cochrane Database of Systematic Reviews (via OVID)**

Keyword search – Search Tools – “Tree”

MeSH Headings

**1**

"geriatric patien*".af. or aging.sh. or ageing.af. or aged.sh. or elder*.af. or geriatric*.af. or "older adul*".af. or "older people".af. or aging.af. or frail elderly.sh.

**2.**
multimorbid*.af. or frailty.sh. or frail*.af. or "multiple comorb*".af. or "multiple chronic conditio*".af. or "frailty syndrome".af.

**3.**

("geriatric rehabilitation" or telerehabilitation or tele-rehabilitation).ab. or telerehabilitation.sh. or "mobile rehabilitation".ab. or "home-based rehabilitation".ab. or "home rehabilitation".ab. or "remote rehabilitation".ab. or "inpatient rehabilitation".ab. or "outpatient rehabilitation".ab. or rehabilitation.ab.

**4.**

1 AND 2 AND 3

**5.**

participation.af. or patient participation.sh. or "patient participation".af. or "rehabilitation process".af. or "care process".af. or "process improvement".af. or "process management".af. or "process evaluation".af. or eligibility determination.sh. or "eligibility determinatio*".af. or "eligibility requiremen*".af. or "eligibility criteria".af. or patient-centered care.sh. or "patient centered".af. or "patient centred".af. or patient-centered.af. or patient-centred.af. or Activities of Daily Living.sh. or "Activities of Daily Living".af. or adl.af. or Quality of Life.sh. or "quality of life".af. or qol.af. or geriatric assessment.sh. or "geriatric assessment".af. or "comprehensive geriatric assessment".af. or "frailty assessment".af. or cga.af. or patient care team.sh. or "patient care team".af. or multidisciplin*.af. or interdisciplin*.af. or multiprofessional*.af. or interprofessional*.af. or "team structure".af. or "healthcare team".af. or "care team".af. or "rehabilitation team".af. or "rehabilitation care team".af. or treatment.af. or intervention.af. or therapy.af. or "treatment process".af. or "patient discharge".af. or "discharge planning".af. or "discharge management".af.

**6.**

continuity of patient care.sh. or "care transition".af. or "patient transfer".af. or "patient transition".af. or "continuity of care".af. or facilitator*.af. or barrier*.af. or assignment.af. or referral.af. or patient admission.sh. or admission.af. or "outcome measure*".af. or "measure*".af. or evaluation.af. or "test batter*".af. or patient reported outcome measures.sh. or "patient reported outcome measures".af. or prom.af. or proms.af. or "patient reported experience measures".af. or prem.af. or prems.af. or goal-setting.af. or "goal setting".af. or Decision Making, shared.sh. or "shared decision making".af. or multicomponent.af. or individualize*.af. or sdm.af. or personalize*.af. or "core team".af. or education.sh. or "outcome assessment".af. or (Outcome and Process Assessment, Health Care).sh. or education.af. or training.af. or health resources.sh. or resource*.af. or Patient Readmission.sh. or readmission.af. or discharge.af. or Mortality.sh. or mortality.af.

**7.**

5 or 6

4 **AND** 7

*Or: 1. AND 2. AND 3. AND 7.*

**507 results**

**EMBASE 1996-2025 (via OVID)**

Keyword search – Search Tools – “Map Term”

Subject Headings

**1.**

"geriatric patien*".af. or aging.sh. or ageing.af. or aged.sh. or elder*.af. or geriatric*.af. or "older adul*".af. or "older people".af. or aging.af.

**2.**

multimorbid*.af. or frailty.sh. or frail*.af. or "multiple comorb*".af. or "multiple chronic conditio*".af. or "frailty syndrome".af.

**3.**

("geriatric rehabilitation" or telerehabilitation or tele-rehabilitation).ab. or telerehabilitation.sh. or "mobile rehabilitation".ab. or "home-based rehabilitation".ab. or "home rehabilitation".ab. or "remote rehabilitation".ab. or "inpatient rehabilitation".ab. or "outpatient rehabilitation".ab. or rehabilitation.ab.

**4.**

patient participation.sh. or participation.af. or "patient participation".af. or "rehabilitation process".af. or "care process".af. or "process improvement".af. or "process management".af. or "process evaluation".af. or eligibility.sh. or "eligibility determinatio*".af. or "eligibility requiremen*".af. or "eligibility criteria".af. or person centered care.sh. or "patient centered".af. or "patient centred".af. or "patient-centered".af. or "patient-centred".af. or daily life activity.sh. or "activities of daily living".af. or adl.af. or quality of life.sh. or "quality of life".af. or qol.af. or geriatric assessment.sh. or "geriatric assessment".af. or "comprehensive geriatric assessment".af. or "frailty assessment".af. or cga.af. or patient care team.sh. or "patient care team".af. or multidisciplin*.af. or interdisciplin*.af. or multiprofessional*.af. or interprofessional*.af. or "team structure".af. or "healthcare team".af. or "care team".af. or "rehabilitation team".af. or "rehabilitation care team".af. or treatment.af. or intervention.af. or therapy.af. or "treatment process".af. or "patient discharge".af. or "discharge planning".af. or "discharge management".af.

**5.**

patient care.sh. or "care transition".af. or "patient transfer".af. or "patient transition".af. or "continuity of care".af. or facilitator*.af. or barrier*.af. or assignment.af. or referral.af. or "patient admission".af. or admission.af. or "outcome measure*".af. or measure*.af. or evaluation.af. or "test batter*".af. or patient-reported outcome.sh. or "patient reported outcome measures".af. or prom.af. or proms.af. or "patient reported experience measures".af. or prem.af. or prems.af. or goal-setting.af. or "goal setting".af. or shared decision making.sh. or "shared decision making".af. or multicomponent.af. or individualize*.af. or sdm.af. or personalize*.af. or "core team".af. or education.sh. or "outcome assessment".af. or "process assessment".af. or education.af. or training.af. or health care planning.sh. or resource*.af. or "patient readmission".af. or readmission.af. or "care need*".af. or discharge.af. or mortality.sh. or mortality.af.

**6.**

4 or 5

**7.**

1 and 2 and 3 and 6

**2592 results**

**Google Scholar**

"rehabilitation process" OR "care process" OR "process improvement" OR "process management" OR "process evaluation" OR "eligibility determination" OR "eligibility requirement" OR "eligibility criteria" OR "patient centred" OR "patient centered" OR "patient centred" OR "patient centered" OR "activities of daily living"

**First 10 pages (100 results)**
